# BRCA1 Recruitment to Transcriptional Pause Sites Is Required for R-Loop-Driven DNA Damage Repair

**DOI:** 10.1016/j.molcel.2015.01.011

**Published:** 2015-02-19

**Authors:** Elodie Hatchi, Konstantina Skourti-Stathaki, Steffen Ventz, Luca Pinello, Angela Yen, Kinga Kamieniarz-Gdula, Stoil Dimitrov, Shailja Pathania, Kristine M. McKinney, Matthew L. Eaton, Manolis Kellis, Sarah J. Hill, Giovanni Parmigiani, Nicholas J. Proudfoot, David M. Livingston

**Affiliations:** 1 Department of Genetics, Harvard Medical School, Boston, MA 02215, USA; 2 Department of Cancer Biology, Dana-Farber Cancer Institute, 450 Brookline Avenue, Boston, MA 02215, USA; 3 Sir William Dunn School of Pathology, University of Oxford, Oxford, OX1 3RE, UK; 4 Department of Biostatistics and Computational Biology, Dana-Farber Cancer Institute, 450 Brookline Avenue, Boston, MA 02215, USA; 5 Department of Biostatistics, Harvard School of Public Health, Boston, MA 02115, USA; 6 Broad Institute of MIT and Harvard, Cambridge, MA 02142, USA; 7 Computer Science and Artificial Intelligence Laboratory (CSAIL), MIT, Cambridge, MA 02139, USA

## Abstract

The mechanisms contributing to transcription-associated genomic instability are both complex and incompletely understood. Although R-loops are normal transcriptional intermediates, they are also associated with genomic instability. Here, we show that BRCA1 is recruited to R-loops that form normally over a subset of transcription termination regions. There it mediates the recruitment of a specific, physiological binding partner, senataxin (SETX). Disruption of this complex led to R-loop-driven DNA damage at those loci as reflected by adjacent γ-H2AX accumulation and ssDNA breaks within the untranscribed strand of relevant R-loop structures. Genome-wide analysis revealed widespread BRCA1 binding enrichment at R-loop-rich termination regions (TRs) of actively transcribed genes. Strikingly, within some of these genes in BRCA1 null breast tumors, there are specific insertion/deletion mutations located close to R-loop-mediated BRCA1 binding sites within TRs. Thus, BRCA1/SETX complexes support a DNA repair mechanism that addresses R-loop-based DNA damage at transcriptional pause sites.

## Highlights

• Endogenous BRCA1 and senataxin (SETX) interact in a BRCA1-driven process
• BRCA1/SETX complexes are recruited to R-loop-associated termination regions (TRs)
• BRCA1/SETX complexes suppress transcriptional DNA damage arising at nearby R-loops
• BRCA1 breast cancers reveal indel mutations near BRCA1 TR binding regions

## Graphical Abstract

**Figure:**
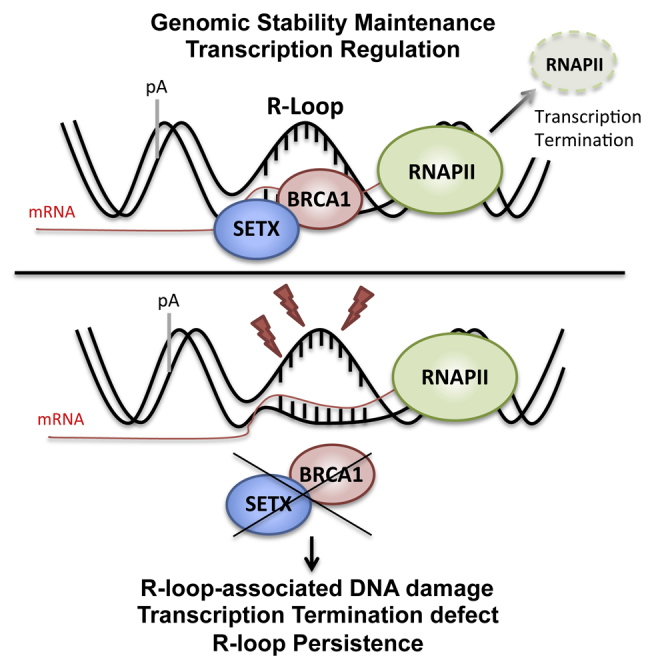

## Teaser

Transcriptional R-loops represent a potential threat to genome integrity. Hatchi et al. show that BRCA1, in partnership with SETX, is engaged in a DNA repair mechanism that deals with R-loop-associated genomic instability at transcriptional termination pause sites.

## Introduction

Germline mutations of the breast cancer susceptibility gene 1 (*BRCA1*) significantly increase the risk of developing breast and ovarian cancers (Tutt and Ashworth, 2002). Since its discovery, researchers have intensively investigated the mechanisms by which full-length BRCA1 (p220) functions as a tumor suppressor.

BRCA1 is engaged in numerous direct and indirect physical interactions with specific partner proteins (Huen et al., 2010). As part of several complexes, BRCA1 contributes to double-strand DNA break (DSB) repair by homologous recombination (HR), stalled fork repair, cell-cycle checkpoint activation, transcription regulation, heterochromatin maintenance, mitotic spindle formation, RNA splicing control, and estrogen metabolism (Gardini et al., 2014, Gorski et al., 2011, Mullan et al., 2006, Pathania et al., 2011, Savage et al., 2014a, Savage et al., 2014b, Venkitaraman, 2014, Zhu et al., 2011). Many of these BRCA1 properties and, in particular, those that protect genome integrity, probably contribute to its tumor suppressing function (Silver and Livingston, 2012, Tutt and Ashworth, 2002). However, in the absence of a coherent underlying mechanism, there is still no definitive evidence of which specific BRCA1 functions are required for breast and ovarian cancer suppression (Huen et al., 2010, Venkitaraman, 2014). Therefore, identifying new BRCA1 binding partners and their associated functions may yield valuable insights.

Our laboratory has identified senataxin (SETX) as a putative BRCA1 binding partner in a yeast two hybrid and several, independent TAP-MS-based genome-wide BRCA1/protein interaction screens (Hill et al., 2014; and D.M.L., unpublished data). Disruption of the *Setx* gene in mice leads to a defect in spermatogenesis, caused by failure of meiotic recombination (Becherel et al., 2013). *Sen1*, the *SETX* yeast homolog, was shown to contribute to the processing of various RNA species and to the distribution of RNA polymerase II (RNAPII) across the genome (Mischo et al., 2011, Steinmetz et al., 2006, Ursic et al., 1997). This probably occurs via its direct interaction with RNAPII and certain RNA processing factors (Suraweera et al., 2009).

While transcription is an essential cellular process, it also represents a potential threat to genome integrity (Kim and Jinks-Robertson, 2012). Several studies indicate that highly transcribed genes exhibit increased rates of mutation and illegitimate recombination (Gaillard et al., 2013). Moreover, a large body of evidence indicates that mutations in certain factors involved at the interface of transcription and RNA processing are associated with genomic instability (Bhatia et al., 2014, Chan et al., 2014, Kleiman and Manley, 1999, Kleiman et al., 2005, Li and Manley, 2006, Stirling et al., 2012). An emerging view is that these mutants contribute to the above-noted phenomena through a common mechanism, which induces the abnormal persistence of co-transcriptional R-loops (three-stranded structures, each consisting of an RNA:DNA hybrid plus the coding strand DNA). Although R-loops are a naturally occurring consequence of transcription and are essential for diverse cellular events (Skourti-Stathaki and Proudfoot, 2014), they can be potentially deleterious to some cellular functions and compromise genome integrity (Aguilera and García-Muse, 2012, Hamperl and Cimprich, 2014). Indeed, unresolved R-loop structures can expose the displaced, coding ssDNA to nicking and/or other forms of damage (Daniel and Nussenzweig, 2013, Wimberly et al., 2013), as well as impair transcription (Aguilera, 2002, Huertas and Aguilera, 2003) and DNA replication fork progression (Gan et al., 2011, Helmrich et al., 2011).

Interestingly, *SETX* is involved in RNAPII transcription termination and resolves R-loops that form at G-rich transcription pause sites (Skourti-Stathaki et al., 2011). It also associates with processing replication forks and facilitates their progression through RNAPII transcribed genes by displacing R-loops (Alzu et al., 2012). In part through its genetic interaction with DNA repair genes involved in HR, senataxin also protects the genome from transcription-associated instability (Mischo et al., 2011, Ursic et al., 2004). Similarly, SETX, by resolving R-loops at sites of transcription and replication collision, is engaged at the interface of replication stress, transcription, and DNA damage (Yüce and West, 2013).

Interestingly, BRCA1-containing complexes restrict DNA damage induced by aberrant transcription or RNA processing via proposed interactions with multiple transcription and RNA processing factors, including RNAPII (Anderson et al., 1998, Bennett et al., 2008, Kawai and Amano, 2012, Kleiman and Manley, 1999, Kleiman et al., 2005, Savage et al., 2014a, Scully et al., 1997).

In view of these associations, we have asked whether BRCA1 plays a significant role in the repair of R-loop-associated DNA damage arising at termination sites. We find that BRCA1 and SETX form a physiological complex, recruited in a BRCA1-dependent manner to a subset of transcription termination pause sites of highly transcribed genes. There they act to suppress co-transcriptional R-loop-associated DNA damage. Unexpectedly, in breast tumor tissues carrying inherited BRCA1 mutations, insertion/deletion somatic mutations were found in the vicinity of BRCA1-bound gene termination sites where BRCA1 normally engages in the repair of R-loop-associated DNA damage.

## Results

### Identification of BRCA1 as a Scaffolding Partner for SETX at the β-actin Transcription Termination Site

**Figure 1:**
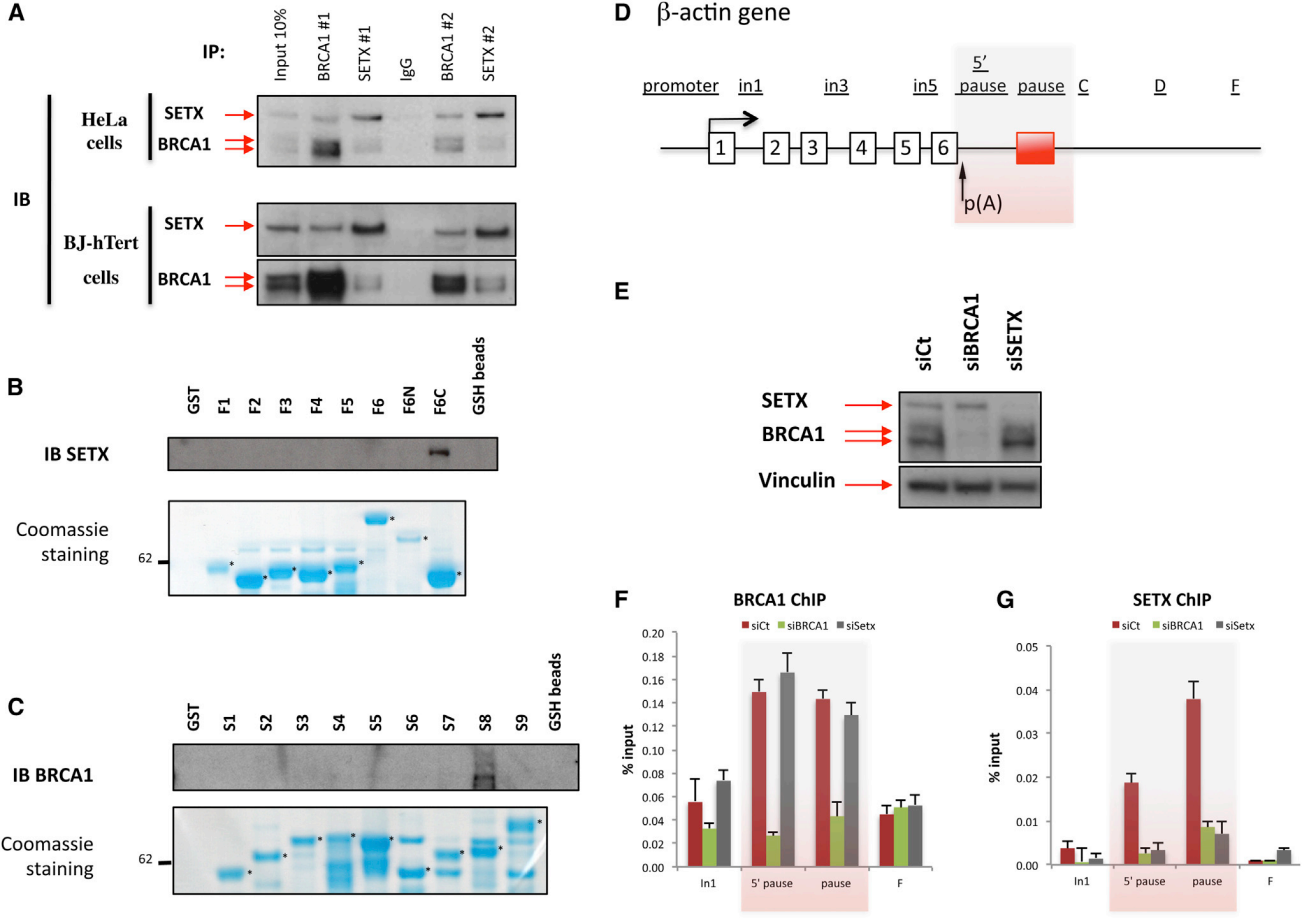
BRCA1 Interacts with SETX and Is Required for SETX Recruitment to the R-Loop-Associated Termination Pause Region of the Human β-actin Gene (A) Co-immunoprecipitation (IP) of endogenous BRCA1 and SETX in HeLa cell (top) and in BJ-hTert human fibroblast extracts (bottom), using antibodies against BRCA1 (BRCA1 #1/#2) or SETX (SETX #1/#2). IgG, negative control. (B) In vitro interaction assay using recombinant GST-BRCA1 fragments performed in HeLa cells. GSH bead-bound proteins were eluted and analyzed by immunoblot. Bottom: relative abundance of each recombinant affinity purified fusion protein (marked with a star) visualized by SDS-PAGE after Coomassie staining. Additional bands are readthrough and/or degradation products of recombinant proteins. (C) Same as in (B) but with recombinant GST-SETX fragments. (D) Schematic representation of the human β-actin gene: exons are numbered, and the red box reflects the existence of a transcription pause element. Location of primers used for the ChIP experiments are depicted above the diagram. (E) Immunoblot that reflects the efficiency of the siRNA-mediated depletion of BRCA1 and SETX. Top: SETX and BRCA1; bottom: Vinculin used as a loading control. (F) ChIP analyses performed on the β-actin gene from mock-treated (red bars), BRCA1- (green bars), or SETX-depleted cells (gray bars) using BRCA1 Ab #3. Average ChIP values ± SD from three independent experiments are shown. (G) Same ChIP experiments as in (F) but with SETX Ab #1. See also Figures S1 and S2.

To investigate whether BRCA1 is involved in R-loop-driven DNA damage responses, we first assessed the physiological relevance of a BRCA1 and SETX interaction, recently identified in our proteomic screens (Hill et al., 2014) and suggested by others (Becherel et al., 2013). Endogenous BRCA1 and SETX co-immunoprecipitation (co-IP) was carried out in HeLa cell extracts, using two, different mono-specific antibodies (Abs) (Figures S1A and S1B). IP of each protein revealed significant co-IP of the other. Irrelevant IgG gave negative results. These results imply the existence of endogenous BRCA1/SETX-containing complexes in these cells (Figure 1A). Weak or undetectable co-IP followed RNAi-driven BRCA1 and SETX depletion, thereby validating Ab specificity (Figure S1C). Two-way BRCA1/SETX co-IP was also apparent in primary, diploid human BJ-hTERT fibroblast extracts (Figure 1A, bottom), suggesting the existence of a physiological interaction between BRCA1 and SETX.

In an effort to map BRCA1 and SETX domains that participate in their interaction, we tested multiple, GST-tagged recombinant fragments that contain various BRCA1 and SETX sequences in glutathione S-transferase (GST) binding assays (Scully et al., 1997, Suraweera et al., 2009) (Figure S1D). The results showed that SETX interacts with F6C, which contains a BRCT (BRCA1 C-terminal) motif (Figure 1B). Intriguingly, F6, which also includes F6C, did not interact with SETX, although F6 was sufficiently folded to interact with another BRCT domain binding protein, BACH1 (Figure S1E) (Cantor et al., 2001). Reciprocal SETX mapping revealed that fragment S8 interacted with BRCA1 (Figure 1C). These results imply that specific structural units of both proteins participate in their interaction. A more extensive analysis will be required to obtain a full picture of this process.

In light of the existence of BRCA1/SETX binding, we tested for BRCA1 co-recruitment at transcription termination sites associated with R-loops by chromatin immunoprecipitation (ChIP) performed on the endogenous human β-actin gene (Figure 1D) (Skourti-Stathaki et al., 2011). Cross-linked HeLa cell chromatin was harvested before and after siRNA depletion of either BRCA1 or SETX. Depletion efficiency was verified at the protein level (Figure 1E).

Specifically, we found that in mock siRNA-transfected cells BRCA1 binding was significantly enriched (3- to 6-fold) at the transcription termination site (5′ pause and pause site probes) when compared to the signals obtained following BRCA1 depletion (Figure 1F). Notably, while BRCA1 depletion suppressed these binding signals, SETX depletion did not. We also confirmed the existence of SETX accumulation at the β-actin transcription termination pause site (Skourti-Stathaki et al., 2011) (Figure 1G). BRCA1 depletion impaired SETX binding to these sites, implying that its recruitment is BRCA1 dependent at this locus. Similar results were obtained when BRCA1 and SETX hairpins were substituted for cognate siRNA species (Figures S2A and S2B). These findings demonstrate a physiological BRCA1/SETX interaction at the β-actin transcription termination site and suggest that BRCA1 acts as an anchor for SETX therein.

### R-Loop Formation at the β-actin Gene Transcription Termination Site Triggers BRCA1/SETX Complex Recruitment

**Figure 2:**
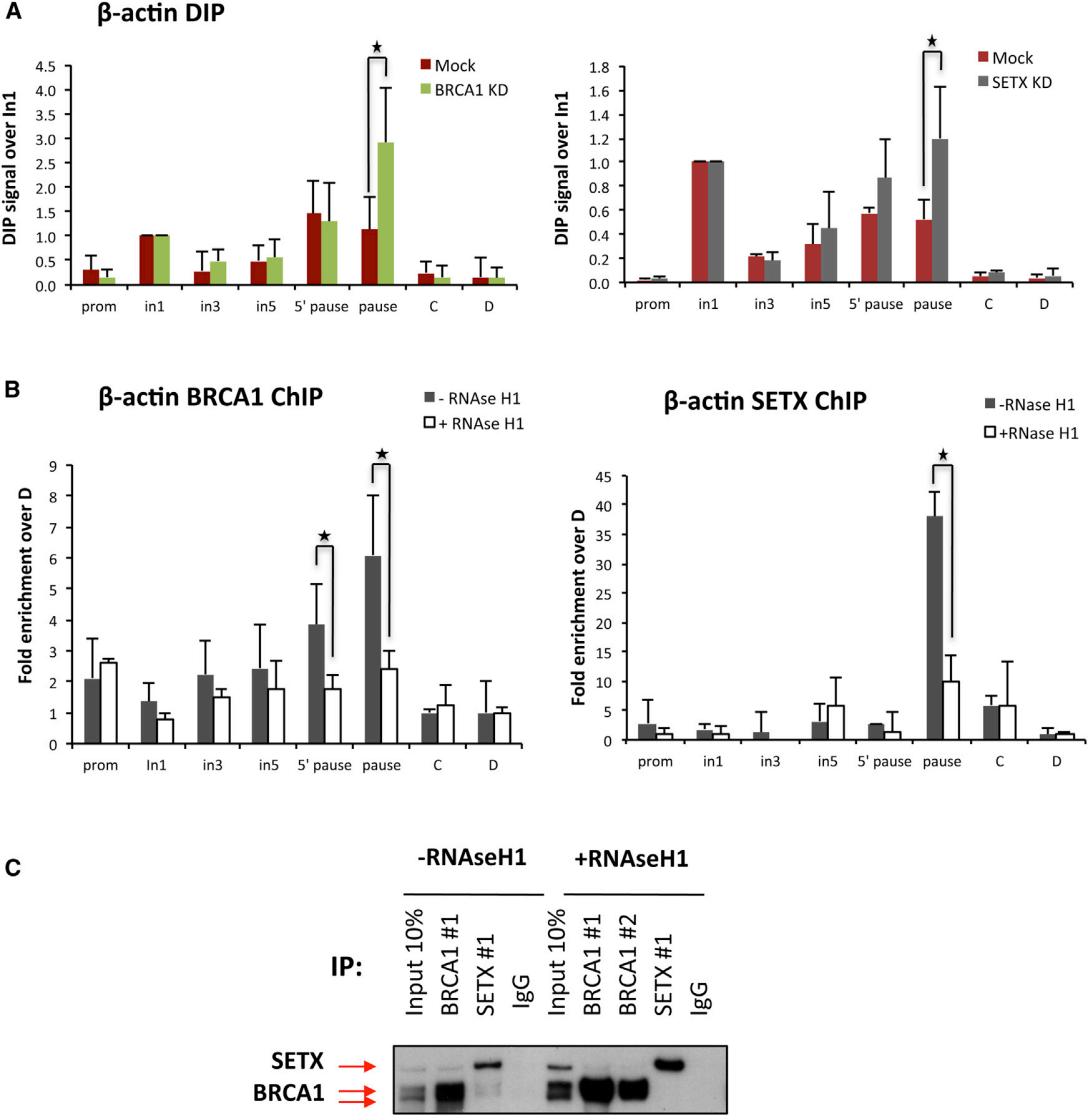
BRCA1 Recruitment to the β-actin Termination Pause Site Is Mediated by R-Loops (A) RNA:DNA immunoprecipitation (DIP) analyses performed in HeLa from mock-treated or BRCA1-depleted cells (BRCA1 KD) (left) and mock-treated or SETX-depleted cells (SETX KD) (right). (B) BRCA1 and SETX ChIP experiment performed in control conditions (−RNaseH1) or with RNaseH1 overexpression (+RNaseH1). Average DIP and ChIP values ± SD from three, independent experiments are shown. ^∗^p < 0.05. (C) BRCA1 and SETX co-IP experiments performed in HeLa cells ± RNaseH1 expression. IgG, negative control.

To search for a connection between the formation of R-loops and the recruitment of BRCA1/SETX at the β-actin termination site, we first asked whether RNA:DNA hybrids forming within this gene are affected by BRCA1 and SETX depletion. We measured R-loop formation over the β-actin gene, employing DNA:RNA hybrid immunoprecipitation (DIP) of uncross-linked DNA using a monoclonal Ab specific for RNA:DNA hybrids (S9.6) (Skourti-Stathaki et al., 2011). As shown in Figure 2A, a modest, but reproducible, increase in R-loop abundance was detected within the β-actin gene pause site, following BRCA1 or SETX depletion. While this result suggests that BRCA1 and SETX affect R-loop formation, it is important to note that BRCA1 depletion also elicited a phenotype suggestive of a transcription termination defect that was previously reported upon SETX depletion. It was manifest by RNAPII accumulation at the pause site (Skourti-Stathaki et al., 2011) (Figure S2C). The recruitment of other RNA:DNA helicases in the absence of SETX to resolve R-loops over these regions, as well as potential differences in transcription levels after BRCA1 and SETX depletion, might explain the lack of a more substantial increase in R-loops levels.

We then tested BRCA1 and SETX chromatin occupancy over the β-actin gene following R-loop suppression. ChIP experiments were performed in HeLa cells before and after overexpression of GFP-tagged RNaseH1 (Figure S3A), which significantly reduced R-loop formation over the β-actin gene (Skourti-Stathaki et al., 2014). In RNaseH1-overexpressing cells, we observed a significant suppression of BRCA1 and SETX binding at the transcription termination site, as compared with control cells (Figure 2B). Moreover, as shown in Figure 2C, RNaseH1 overexpression led to a reduction in the co-IP signal, implying that BRCA1/SETX complex formation at these genomic loci is mediated by R-loops.

Thus, our results suggest that BRCA1/SETX complexes bind at termination region (TR) sites in response to local R-loop formation and that they also suppress R-loop abundance, in part, by participating in the regulation of transcription and of transcription termination.

### BRCA1/SETX Complexes Protect DNA at R-Loop-Associated Termination Pause Sites from the Development of ssDNA Breaks

**Figure 3:**
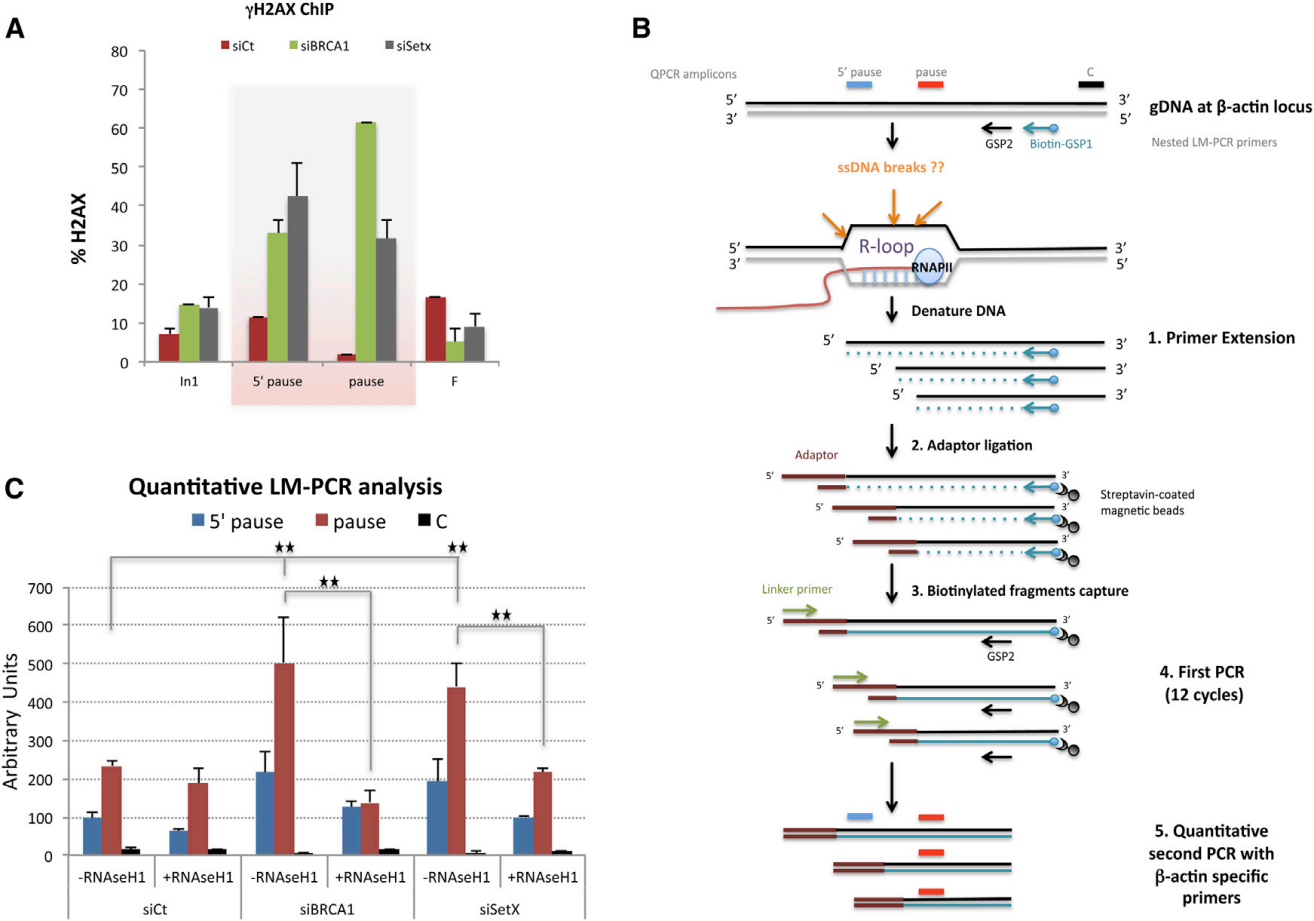
BRCA1/SETX Complex at the β-actin Pause Site Protects Cells from R-Loop-Driven ssDNA Breaks (A) ChIP analysis performed on the β-actin gene as in Figure 1 using γH2AX and total H2AX antibodies. Histograms represent the proportion of total H2AX phosphorylated on Ser139 (i.e., γH2AX). Average ChIP values ± SD from three independent experiments are shown. (B) LM-PCR strategy used to identify R-loop-associated ssDNA breaks on the coding strand. See Supplemental Experimental Procedures. (C) Quantitative detection of ssDNA after LM-PCR performed on the β-actin gene before and after BRCA1 or SETX knockdown and with or without ectopic RNaseH1 expression. QPCR values are average ± SD from three independent experiments. ^∗^p < 0.05, ^∗∗^p < 0.007 by one-tailed Student’s t test. See also Figure S3.

Since R-loop accumulation can increase the risk of genomic instability by promoting DNA damage (Hamperl and Cimprich, 2014, Skourti-Stathaki and Proudfoot, 2014), we tested whether BRCA1 and/or SETX depletion triggers the generation of DNA damage in the vicinity of persistent R-loops. Specifically, γH2AX ChIP assays were performed over the β-actin gene. After depletion of either BRCA1 or SETX, we observed significantly increased signals of γH2AX largely restricted to the termination region (Figure 3A). This implied that loss of BRCA1/SETX complexes over the R-loop-associated pause site contributed to the accumulation of local damage.

Since several studies suggest that the ssDNA of an R-loop is prone to transcription-associated mutations or DNA breaks (Aguilera and García-Muse, 2012, Wimberly et al., 2013), we adapted a ligation-mediated quantitative PCR (LM-qPCR) approach to test whether ssDNA breaks had occurred on the non-template strand following BRCA1 or SETX depletion (Figure 3B). Briefly, we ligated an adaptor to putative ssDNA ends after performing a primer extension reaction over the far 3′ end of the β-actin gene. The goal was to amplify any post-ligation DNA fragments that harbor both the adaptor sequence and a segment of the 3′ end of the β-actin gene.

In the absence of BRCA1 or SETX, significant signals were observed when using 5′ pause and pause site primers, thereby reflecting the existence of one or more ssDNA breaks located near the termination region (Figures 3C and S3B). The 3′ R-loop-free C region (Figure 2A) was used as a negative control to validate the specificity of the above-noted results. We also tested the anti-sense strand and failed to observe LM-qPCR signals above background following BRCA1 or SETX depletion (Figure S3C). This suggested that the observed breaks affected only the non-template strand.

We then asked whether RNaseH1 overexpression suppressed the appearance of ssDNA breaks in the β-actin gene in BRCA1- or SETX-depleted cells. The background signals in control region C remained barely detectable, while RNaseH1-expressing BRCA1- or SETX-depleted cells revealed a significant signal decrease within the 5′ pause and pause regions (Figures 3C and S3B). Taken together, these data argue that, in the β-actin gene, BRCA1 and SETX are involved in the prevention/repair of ssDNA breaks arising within the coding strand R-loop near their 3′ end binding site(s).

### BRCA1/SETX Complexes Form at Several R-Loop-Associated Genomic Loci and Protect Cells from the Development of ssDNA Breaks

**Figure 4:**
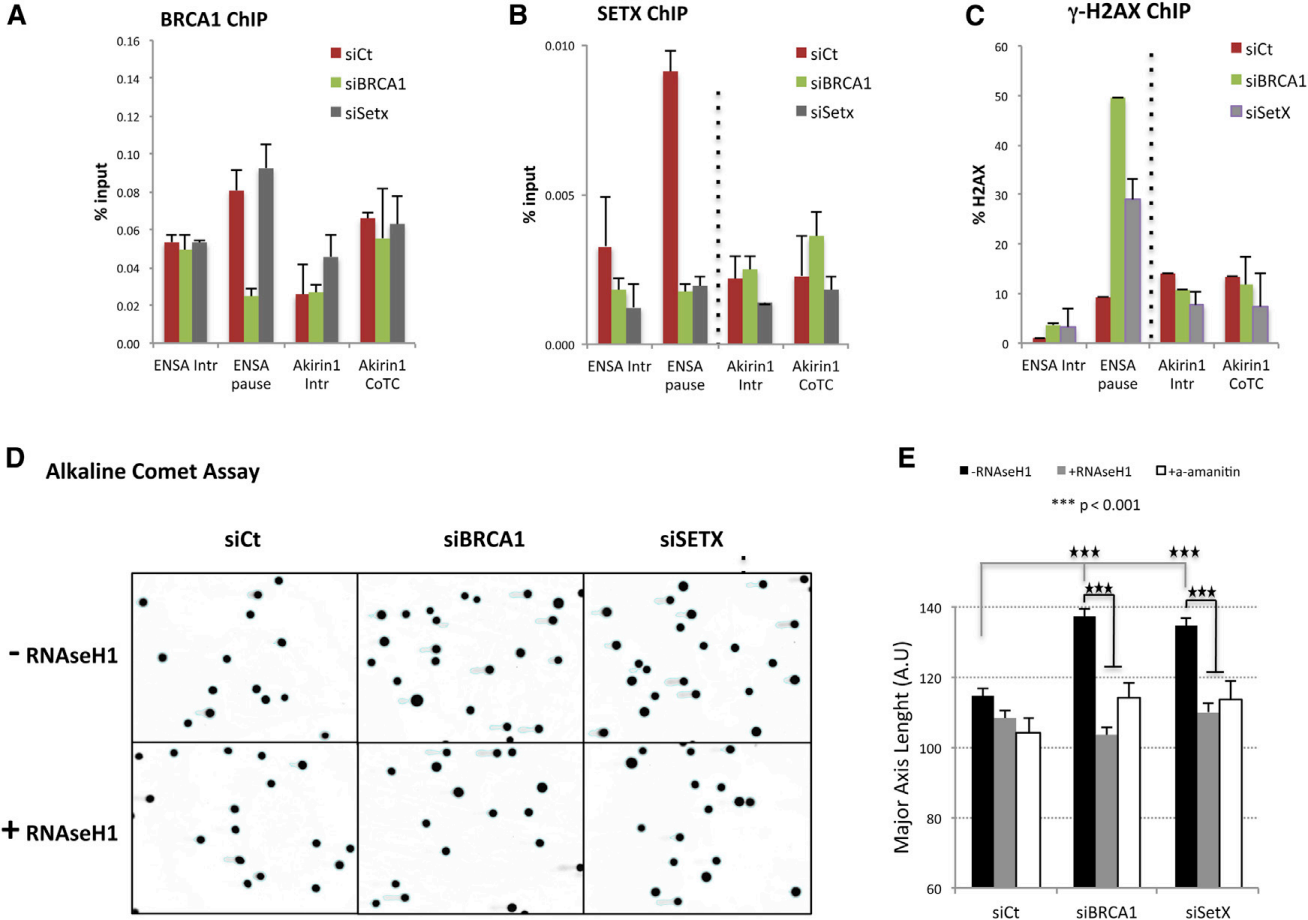
DNA Damage Arising in Absence of BRCA1/SETX Complexes at Termination Pause Sites Is R-Loop Dependent (A and B) BRCA1 (A) and SETX (B) ChIP analyses performed on the ENSA and Akirin1 genes in HeLa cells transfected with siCt, siBRCA1, or siSETX. ENSA and Akirin1 transcription termination is regulated by R-loops and CoTC sequences, respectively. Intronic regions (Intr) were studied as controls. Average ChIP values ± SD from three independent experiments are shown. (C) γH2AX ChIP experiments performed as in (A) and analyzed as in Figure 3A. Average ChIP values ± SD from three independent experiments are shown. (D) Representative pictures of comet assays performed under alkaline conditions in HeLa cells transfected with siCt, siBRCA1, or siSETX in the absence (−RNaseH1) and presence (+RNaseH1) of ectopic RNaseH1 expression. (E) Quantitative analysis of comet tail lengths for each condition showed in (D). Average tail lengths ±SEM from three independent experiments are shown. ^∗∗∗^p < 0.001 by two-tailed Student’s t test. See also Figure S4.

To search for a global effect of BRCA1/SETX function, we first searched for evidence within two gene sets. On one hand, we analyzed two genes (*ENSA*, *Gemin7*) in which transcription termination is also dependent on R-loops (Skourti-Stathaki et al., 2014). In both genes, BRCA1 and SETX binding was enriched at the relevant transcription termination regions as compared to an irrelevant intronic segment (Figures 4A, 4B, S4A, and S4B). Although the BRCA1 antibody used in ChIP experiments displayed some background signal, its specificity was confirmed by its sensitivity to BRCA1 depletion. In parallel, we studied the *Akirin1* and *cyclinB1* genes in which transcription termination is regulated by an R-loop-free co-transcriptional cleavage (CoTC) mechanism (Nojima et al., 2013, Skourti-Stathaki et al., 2014, White et al., 2013). BRCA1 and SETX ChIP analysis revealed no specific signals in either intronic region or the CoTC regions of *Akirin1* and *cyclinB1*, reinforcing the hypothesis that BRCA1/SETX recruitment requires R-loop formation (Figures 4A, 4B, S4A, and S4B).

We also examined the DNA damage signaling response in these two categories of genes by searching for γH2AX signals over the termination regions using ChIP qPCR. Similar to *β-actin*, increased γH2AX signals were clearly visible over the pause element of both *ENSA* and *Gemin7* following BRCA1 or SETX depletion, but not over the control intronic region (Figures 4C and S4C). By contrast, no γH2AX enrichment was detected at “R-loop-free” CoTC termination regions within *Akirin1* or *cyclinB1*. These results are consistent with our β-actin ChIP data (Figure 3A) and with the presence of local γH2AX signals in the absence of BRCA1/SETX complexes over *ENSA* and *Gemin7*. Thus, they can be viewed as a reflection of local R-loop-associated DNA damage. These findings imply that BRCA1/SETX complexes participate in the repair or prevention of this form of damage in these two genes bearing R-loop-associated transcription termination regions.

We then asked whether BRCA1/SETX function at R-loop-forming termination sites is a genome-wide phenomenon. First, comet assays were performed to search for widespread DNA breaks as a consequence of BRCA1 or SETX depletion. As predicted, under alkaline conditions, which detect both ssDNA breaks and DSBs, we observed a significant increase in comet tail lengths in both BRCA1- and SETX-depleted cells. Moreover, RNaseH1 overexpression in both settings decreased the abundance of cells with comets as well as their length (Figures 4D and 4E). Similar results were observed when the cells were treated with a low dose of α-amanitin that acts to inhibit RNAPII elongation. These results confirm that R-loop structures, as by-products of transcription, create numerous fragile genomic sequences prone to BRCA1/SETX depletion-associated damage. Although we also observed an increase in comet tail lengths in BRCA1- and SETX-depleted cells assayed in neutral buffer conditions (probably reflecting DSB), these DNA damage phenotypes did not revert after overexpression of RNaseH1 or exposure to α-amanitin (Figure S4D). Thus, R-loop-dependent DNA damage following BRCA1/SETX depletion is mainly composed of ssDNA breaks.

### BRCA1 Is Globally Associated with Transcription Termination Sites of Highly Transcribed Genes

**Figure 5:**
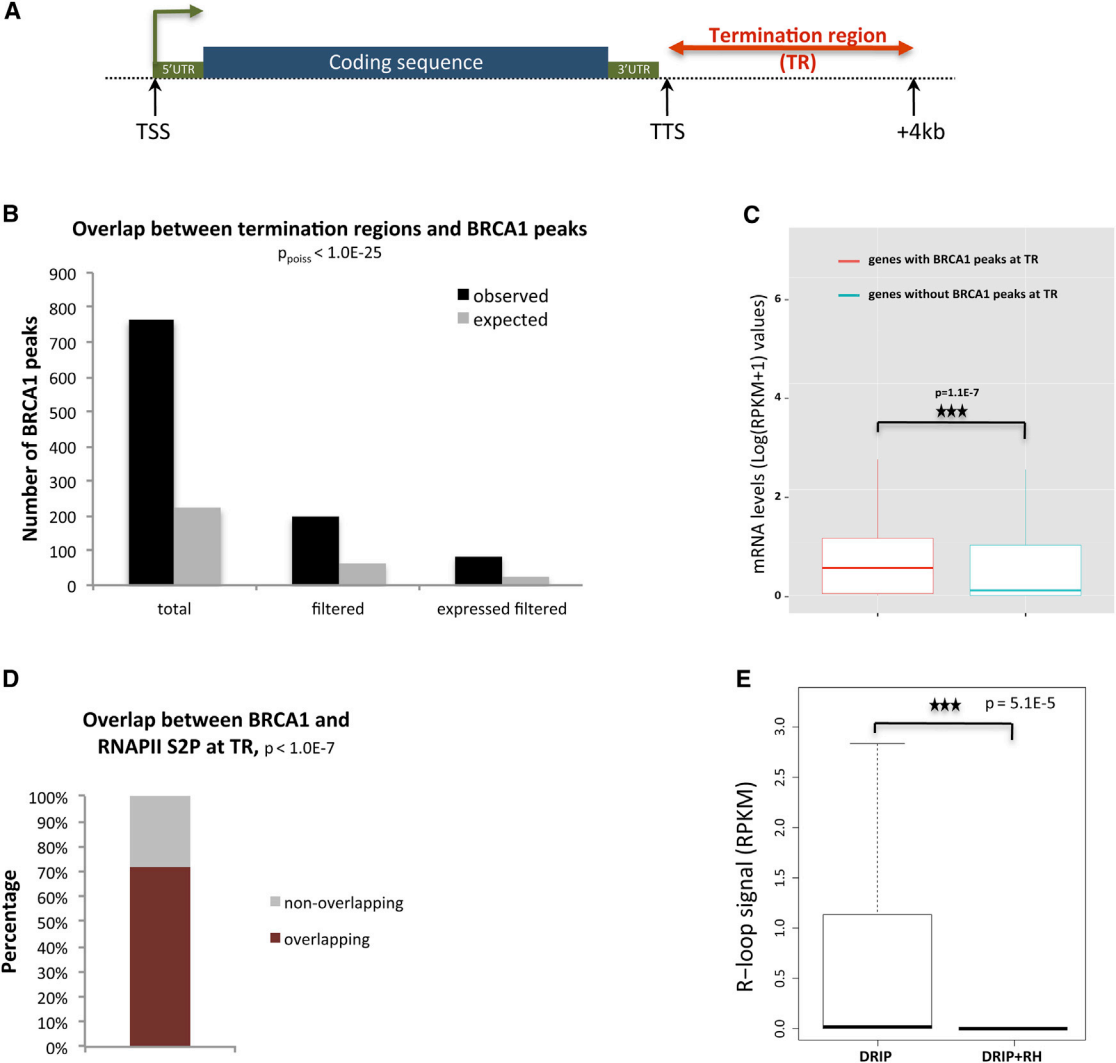
BRCA1 Binds the Transcription Termination Region of a Substantial Subset of Actively Expressed Mammalian Genes (A) Diagram of candidate BRCA1 TR binding regions. Putative termination regions (TRs) were defined as segments extending from the TTS to TTS + 4 kb. (B) Total number of observed and expected overlaps between TR and BRCA1 peaks (p_poiss_ = 2.2E-177). “Filtered”: BRCA1 ChIP-seq peaks divested of those overlapping promoter regions and transcripts (p_poiss_ = 1.2E-42). “Expressed filtered”: BRCA1 peaks present in TR region of expressed genes (p_poiss_ = 5E-24). (C) Gene expression comparison between genes with (red) and without (blue) BRCA1 bound to relevant TR. Boxplots reflect the median (50^th^ percentile) of mRNA expression. ^∗∗∗^p = 1.1E-7, Mann-Whitney test. Outliers have been omitted from the plot. (D) Overlap between BRCA1 TR and RNAPIISer2P peaks, overlap enrichment over random (^∗∗∗^p < 1.0E-7), see Experimental Procedures. (E) Boxplots showing DRIP-seq signals (RPKM) of DRIP samples compared with DRIP+RH controls (treated with RNaseH1) in BRCA1 TR candidate regions, ^∗∗∗^p = 5.101E-5, paired Wilcoxon test. See also Figure S5 and Table S1.

In light of the possibility that R-loop-associated termination regions are characteristics of larger numbers of genes, we performed a meta-analysis from several deep-sequencing datasets to search for genomic co-localization of BRCA1 peaks at transcription termination regions. We used the ENCODE BRCA1 ChIP-seq dataset to identify candidate genes regulated by this mechanism (Consortium, 2012). For each gene, a candidate transcription TR was defined as a 4 kb segment downstream of the transcription termination site (TTS) (Figure 5A).

Initially, 764 distinct BRCA1 peaks significantly overlapping a genomic TR were identified. Since BRCA1 is also associated with a subset of promoters (K.M.M. et al., unpublished data) (Gardini et al., 2014), we further filtered these data to exclude any peaks overlapping promoter regions and their associated transcripts. In this filtered gene set, 196 BRCA1 peaks, each associated with a TR, corresponded to 184 genes that are referred to here as BRCA1 TR genes (Figure 5B and Table S1).

Since the DNA damage that developed over R-loop-associated TRs upon BRCA1/SETX depletion is proposed to be R-loop and, therefore, transcription dependent, we analyzed the expression levels of these BRCA1 TR genes. RNA-seq data were used to analyze HeLa cell transcriptomes available from ENCODE. The expression profiles of genes whose filtered TRs did or did not overlap BRCA1 ChIP-seq peaks were compared. The data showed that BRCA1 TR genes clearly displayed higher mRNA levels (p = 1.1E-7), when compared to genes whose termination sites are not associated with a BRCA1 binding site (Figures 5C and S5 and Table S1). These results indicate that BRCA1 TR genes are more highly expressed than those lacking BRCA1 TR binding peaks.

To explore further whether BRCA1 binding at TR is associated with transcription termination, we searched for evidence of the co-localization at termination sites of BRCA1 peaks and RNAPII paused at the 3′ end of the relevant genes (RNAPII S2P) (Davidson et al., 2014). Here we used ENCODE RNAPII CTD Ser2-P ChIP-seq data (Consortium, 2012). This analysis showed that more than 70% of BRCA1 peaks within TRs overlap paused RNAPII (Figure 5D and Table S1), suggesting that BRCA1 is actively engaged in transcription-associated events at termination sites. To investigate whether BRCA1 TR are associated with R-loops, we compared them with the RNA:DNA IP database (DRIP-seq) generated with cells before and after RNaseH1 treatment (DRIP+RH) (Ginno et al., 2012). We found that BRCA1 TR peaks show a significant enrichment of DRIP signal as compared to DRIP+RH (Figure 5E), implying the existence of R-loop formation at the BRCA1-associated TR loci.

These results suggest that in mammalian cells BRCA1 binds to TR (associated with paused RNAPII) in a substantial subset of highly transcribed genes, whose pause sites reflect a strong tendency to form R-loops. Taken together, our findings indicate that BRCA1 and SETX participate in the prevention/repair of ssDNA damage occurring at specific regions (TRs) in response to the transcription-associated formation of R-loops in physiological conditions. Conceivably, these genes require BRCA1/SETX-dependent DNA damage surveillance to ensure regulation of transcription (Huppert et al., 2008, Skourti-Stathaki and Proudfoot, 2014).

### BRCA1 Mutant Breast Cancers Reveal Genomic Alterations at BRCA1-Associated Termination Sites

We next asked whether a defect in the newly detected BRCA1/SETX function could participate in the pathogenesis of BRCA1-deficient breast cancer tumors. Recent large-scale DNA sequencing screens performed in various human cancers have shed light on the nature and location of associated somatic mutations (Alexandrov et al., 2013, Pleasance et al., 2010). We performed a mutation analysis in the 184 BRCA1 TR genes, using the complete catalog of somatic mutations obtained from the whole-genome sequencing of 21 breast cancers, a subset of which (n = 5) were BRCA1 mutant (Nik-Zainal et al., 2012). This catalog includes single base mutations (SBMs), insertions and deletions (indels), and chromosomal rearrangements. For each patient, the genome of healthy/normal mammary tissue was also sequenced, and any mutations therein were deleted from the patient mutation analysis. Since R-loops can be long, we first included promoter regions in this search. More specifically, we first defined for each BRCA1 TR gene a whole gene region of special interest from TSS − 1,250 bp to TTS + 5 kb and searched for BRCA1-specific mutations.

**Figure 6:**
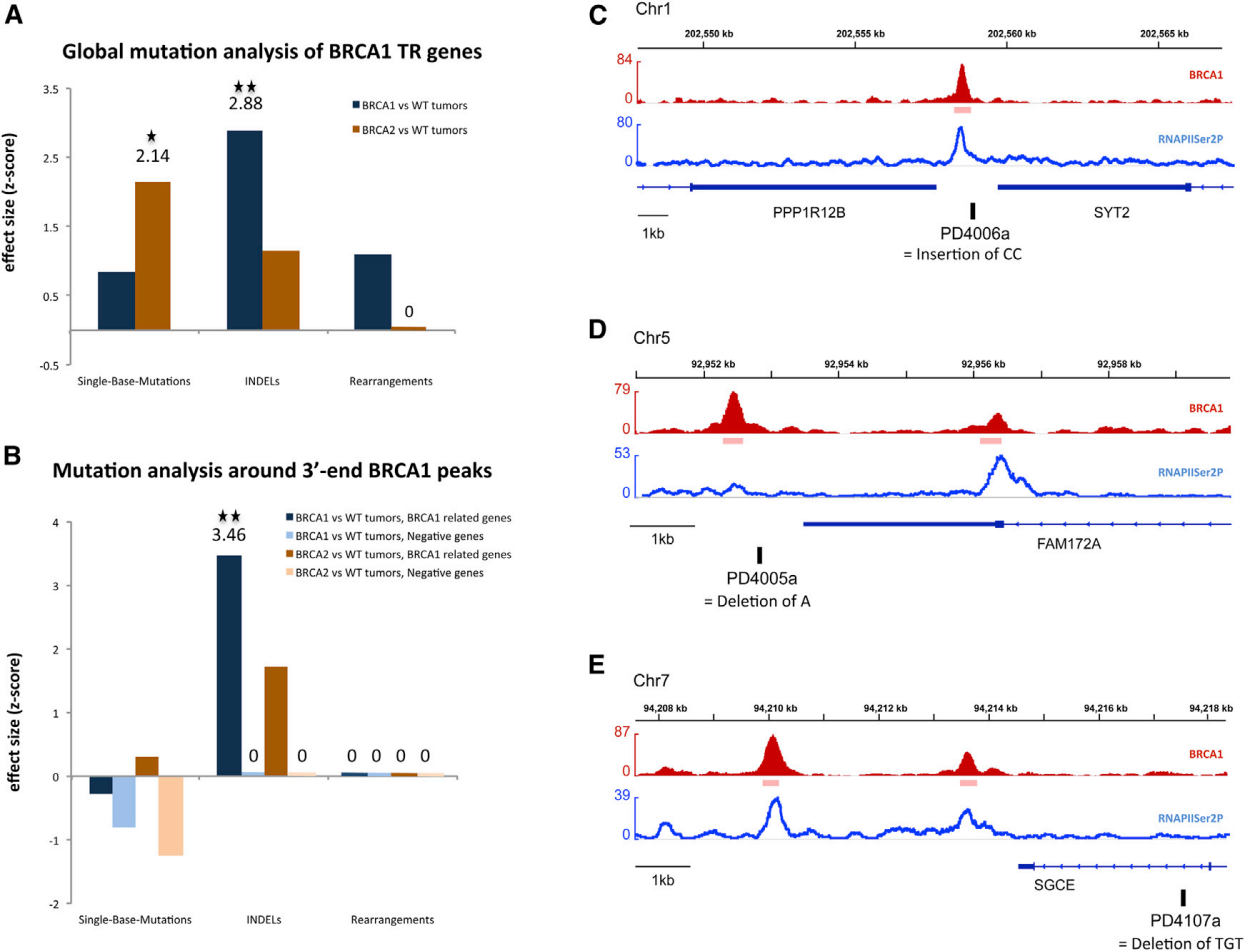
BRCA1-Deficient Breast Cancers Reveal Genomic Abnormalities at and near BRCA1-Associated Termination Sites (A and B) Global mutational analysis carried out in the 184 BRCA1 TR genes using the complete whole-genome catalog of somatic mutations from 21 breast cancers (Nik-Zainal et al., 2012). Effect size comparison (one-tailed CMH *Z* score) between the different tumor subgroups when testing the region from TSS − 1,250 bp to TTS + 5 kb (A) or ± 4 kb from TTS (B). WT tumors = non-BRCA1/BRCA2, and negative genes = CoTC genes. Statistical significant: ^∗^p < 0.05 and ^∗∗^p < 0.01. (C–E) ChIP-seq profiles of BRCA1 (red) and RNAPIISer2P (blue) in BRCA1 TR genes and location of indels (black boxes). Chr, chromosome. See also Figure S6 and Table S2.

We divided the 21 patient datasets into 3 breast cancer types: 12 sporadic (i.e., tumors WT/WT for both BRCA1 and BRCA2), 5 BRCA1, and 4 BRCA2 mutant tumors. Figure 6A compares each of the mutant groups to WT, in search of a quantitative measure of any difference in mutation rate (i.e., effect size) between any 2 groups. The effect size was computed as a standardized difference in mutation counts when all selected genes are considered together. It was obtained separately for each type of mutation analyzed. The results clearly indicate that, within the BRCA1 TR gene regions of interest, there was significant enrichment for indels only in the mutant BRCA1 tumors (z = 2.875, p = 0.009), despite the fact that more indels were detected throughout the entire genomes of BRCA2 than BRCA1 breast cancers (Nik-Zainal et al., 2012). By contrast, these genomic loci were enriched for SBM in the BRCA2 tumors (z = 2.144, p = 0.021). Of note, no significant enrichment for rearrangements was observed. These results indicate that, within BRCA1 TR genes, distinct mutational patterns exist that separate BRCA1 and BRCA2 tumors. This suggests that any biochemical defects that the absence of these proteins elicits may, at least in part, be different.

In parallel, we also performed a global mutation analysis among the “R-loop free” CoTC-regulated genes (“negative” genes) (Nojima et al., 2013) where BRCA1 is not recruited (Figure 4A). We failed to detect any significant change in the species of mutations detected in either BRCA1 or BRCA2 null tumors (Figure S6).

To further investigate the mutational profile at the 3′ ends of the BRCA1 TR genes, we focused on a narrower region defined as: TTS ± 4 kb. Strikingly, the only significant enrichment observed was for indels in BRCA1 mutant tumors (z = 3.464, p = 0.002) (Figure 6B). Precise mapping of the indels in the BRCA1 TR genes in the BRCA1 null tumors indicated that 3 out of 6 were located ∼300–400 bp from the BRCA1 TR peak; 2 were located further away (last intron and ∼1.8 kb downstream of the BRCA1 peak); and another was close to the promoter region (Figures 6C–6E and Table S2). The absence of SBM enrichment in the 3′ end regions of the TR genes in BRCA2 mutant carriers suggests that most of it accumulates at 5′ ends and within the gene body. Of note, none of the few mutations detected in the CoTC genes was located in 3′ end regions.

Overall, these results show that BRCA1-deficient tumors are significantly enriched for indels in BRCA1 TR genes compared to BRCA2 and sporadic breast cancers. A significant fraction of the BRCA1 tumor-associated indels lie in the vicinity of BRCA1 binding TR loci, supporting the view that BRCA1 plays a critical role in preventing/repairing R-loop-mediated damage in the vicinity of R-loop-associated TR.

## Discussion

In this study, a combination of biochemical, molecular, and genetic data provides evidence that a newly identified BRCA1/SETX complex is required to restrain the development of R-loop-mediated DNA damage at specific genomic regions. In particular, we have delineated a sequence of events that is predicted to occur over a substantial subset of R-loop-associated transcriptional pause sites and, when impaired, results in the BRCA1 TR-associated indel mutations observed in BRCA1^−/−^ breast cancer genomes.

Under physiological conditions, R-loops play a role in transcription termination at G-rich pause sites, like those in *β-actin*, *Gemin7*, and *ENSA* (Skourti-Stathaki et al., 2014). They are also present at promoter regions where there is a region of GC-skew that ensures the protection of vicinal CpG island sequences against DNA methylation (Ginno et al., 2012). Though these structures represent R-loops associated with distinct biological functions, they are, nonetheless, still predicted to induce DNA damage (for review see Skourti-Stathaki and Proudfoot, 2014).

How BRCA1 recognizes R-loop structures remains to be determined. Current knowledge focuses mainly upon the factors that regulate their formation (Chan et al., 2014, Hamperl and Cimprich, 2014, Skourti-Stathaki and Proudfoot, 2014). However, little is known about potential direct interaction between specific proteins and R-loops. Several possibilities exist. For example, there could be direct recognition of an RNA:DNA hybrid, e.g., as occurs with the hybrid-binding domain (HBD) of RNaseH1 (Cerritelli and Crouch, 2009), and/or of the non-template ssDNA. For BRCA1, it is also possible that the branched structure of an R-loop resembles the DNA flaps, branched DNA, and/or four-way junctions for which BRCA1 exhibits an intrinsic affinity (Paull et al., 2001). Alternatively, BRCA1 recruitment to R-loops could be mediated by other protein complexes that normally interact with these special DNA structures. These could be components of the RNAPII holoenzyme, splicing machinery, and/or specific chromatin remodeling complexes that are associated with R-loops (Bochar et al., 2000, Boulé and Zakian, 2007, Savage et al., 2014a, Scully et al., 1997, Yarden and Brody, 1999).

Results presented here show that a deficiency in BRCA1/SETX function results in unrepaired ssDNA breaks on the non-template strand at certain R-loop-associated TRs. γH2AX accumulation was observed by ChIP, although the extent of its genomic presence appears to be more restricted than observed at sites of DSB (Paull et al., 2000). It is tempting to speculate that the restricted presence of γH2AX signals is a reflection of the presence of ssDNA breaks as the source of DNA damage and/or that γH2AX spreading is antagonized by high levels of transcription (Iacovoni et al., 2010).

The above considerations aside, how DNA damage occurs at such sites remains unknown. Multiple molecular mechanisms may foster or contribute to the DNA damage associated with R-loops. Superhelical stress and G4 and/or flap endonucleases could generate local ssDNA breaks, some of which could devolve into DSB (Hamperl and Cimprich, 2014). In addition, genome-wide sequencing of human cancers suggests that non-random, clustered mutations may be concentrated in long ssDNA regions, some of which might form R-loop structures (Roberts et al., 2012). Indeed, during transcription, possibly due to the relative chemical susceptibility to damage of ssDNA, there is an increase in the mutation rate associated with the activity of editing enzymes like activation-induced cytosine deaminase (AID) or apolipoprotein B mRNA-editing catalytic polypeptide proteins (APOBEC) (Alexandrov et al., 2013, Beale et al., 2004, Chan et al., 2012). Recently, the latter have been suggested to play a role in the mutational processes that affect breast cancer genomes (Burns et al., 2013, Nik-Zainal et al., 2012, Nik-Zainal et al., 2014).

Taken together, our findings show that BRCA1 contributes to the control and/or repair of R-loop-mediated DNA damage at specific sites, which is, potentially, a significant contributor to the maintenance of genomic stability. Similarly, the implications of a proposed role for BRCA2 in transcription-associated recombination (TAR) and in the processing of R-loops in partnership with RNA processing factors suggest that these structures are a source of cancer-related instability (Bhatia et al., 2014, Gallardo et al., 2003, Huertas and Aguilera, 2003, Savolainen and Helleday, 2009).

*BRCA1*- and *BRCA2*-mutant cancers exhibit differences in histopathology, gene expression profiles, and clinical course, even though they share similarities in their marked levels of genomic instability (Nik-Zainal et al., 2012). By contrast, our observations imply that, at certain transcription termination regions, the mutational signatures of BRCA1 and BRCA2 null tumors are different. Conceivably, these differences arise from the possibility that BRCA1 and BRCA2 respond to/interact with R-loops differently and even in different contexts. Additional whole-genome sequencing from greater numbers of BRCA mutant cancers would be required to address these possibilities in the future. Further studies will also be needed to determine whether such BRCA1/BRCA2 mutational differences contribute directly or indirectly to the biological differences between BRCA1 and BRCA2 breast cancers.

## Experimental Procedures

For detailed experimental procedures, see Supplemental Experimental Procedures.

### Co-Immunoprecipitation Analysis

Whole-cell extracts (WCE) were prepared as previously described (Tardat et al., 2010), except that the lysis mixture was sonicated with a Sonic Dismembrator for 15 s at an amplitude of 20% (Fisher Scientific, Model 120). 200 μg of WCE were incubated for 2 hr at 4°C with anti-BRCA1#1 (SG11, mouse monoclonal), anti-BRCA1#2 (MS110, mouse monoclonal), anti-SETX#1 (A301-105A, Bethyl), anti-SETX#2 (A301-104A, Bethyl), or control IgG. Immune complexes were collected with protein A/G magnetic beads (Dynabeads, Invitrogen) for 15 min at 4°C and washed with IP buffer (with 1 mM DTT) and increasing concentrations of KCl (50/100/150 mM KCl). Bound proteins were eluted in LDS sample buffer 1× (Invitrogen) and analyzed by immunoblotting.

### qPCR ChIP and DIP Experiments

ChIP experiments on BRCA1, SETX, and γH2AX were performed using a modified version of N.J.P. laboratory’s protocol (Skourti-Stathaki et al., 2011), as detailed in the Supplemental Experimental Procedures. DNA:RNA hybrid precipitation (DIP) analysis was performed with the specific monoclonal RNA:DNA hybrid Ab (S9.6) as described in Skourti-Stathaki et al. (2011). Sequences of the DNA primers are listed in Supplemental Experimental Procedures.

### Mutational Analyses of Breast Cancers

Detailed description of the computational and statistical analyses is available in Supplemental Experimental Procedures.

The integrative Genomics Viewer (IGV) genome browser was used to visualize the indels and the BRCA1 and RNAPIISer2P ChIP-seq profiles across the termination regions of the BRCA1 TR genes (Thorvaldsdóttir et al., 2013).

## Author Contributions

S.J.H. and D.M.L. originally suggested and first conceived the rationale for this project. E.H. designed the overall project with help from K.S.-S., K.M.M., D.M.L., and N.J.P. E.H. performed the experiments and analyzed the experimental data unless otherwise stated. E.H. received advice, reagents, and help for the ChIP experiments and the co-immunoprecipitation from K.S.-S., K.K.M., S.P., and S.D. K.S.-S. performed and analyzed the BRCA1 and SETX DIP and part of BRCA1 and SETX ChIP experiments. A.Y., M.L.E., and M.K. performed the genomic analysis of BRCA1 ChIP-seq data and K.K.-G. the meta-analyses correlating RNAPII and DRIP datasets. S.V., L.P., and G.P. performed the global mutational analyses of the breast tumors. E.H. and D.M.L. wrote the manuscript. All authors contributed to the editing of the manuscript.

## Supplemental Information

Document S1. Supplemental Experimental Procedures and Figures S1–S6

Table S1, Related to Figure 5. List of BRCA1 Peaks at Termination Regions List of BRCA1 ChIP-seq peaks identified within termination regions (TRs) showing genomic information: chromosomes, coordinates, strand, gene name associated, RNA-seq RPKM values, and overlap with RNAPIISer2P ChIP-seq peaks.

Table S2, Related to Figure 6. List of the Indels Identified within the BRCA1 TR and CoTC Genes BRCA1 TR and CoTC genes were tested for mutations within 21 breast cancers containing BRCA1 or BRCA2 germline mutations. Genomic information of the indels identified is shown.

Document S2. Article plus Supplemental Information
