# Supplementary material for: BRCA1 Recruitment to Transcriptional Pause Sites Is Required for R-Loop-Driven DNA Damage Repair
Source: Mol Cell. 2015 Feb 19;57(4):636–47. doi: 10.1016/j.molcel.2015.01.011 (PMC4351672; doi:10.1016/j.molcel.2015.01.011)
Supplement: Document S1. Supplemental Experimental Procedures and Figures S1–S6 [file mmc1.pdf]

Molecular Cell

Supplemental Information

# BRCA1 Recruitment to Transcriptional Pause Sites Is Required for R-Loop-Driven DNA Damage Repair

**Elodie Hatchi, Konstantina Skourti-Stathaki, Steffen Ventz, Luca Pinello, Angela Yen, Kinga Kamieniarz-Gdula, Stoil Dimitrov, Shailja Pathania, Kristine McKinney, Matthew L. Eaton, Manolis Kellis, Sarah J. Hill, Giovanni Parmigiani, Nicholas J. Proudfoot, and David M. Livingston**

# Hatchi et al. Figure S1, related to Figure 1

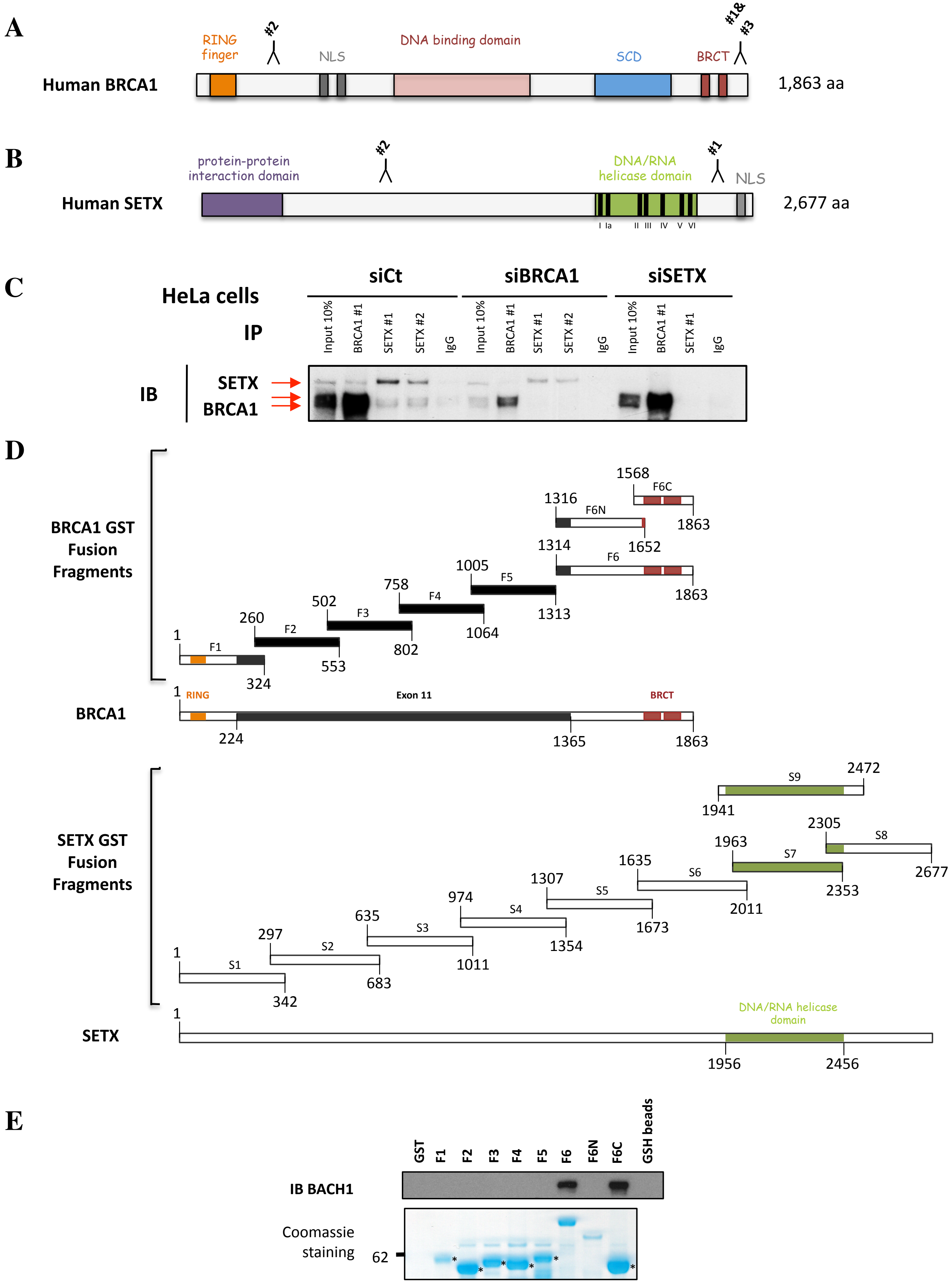

Hatchi et al. Figure S2, related to Figure 1

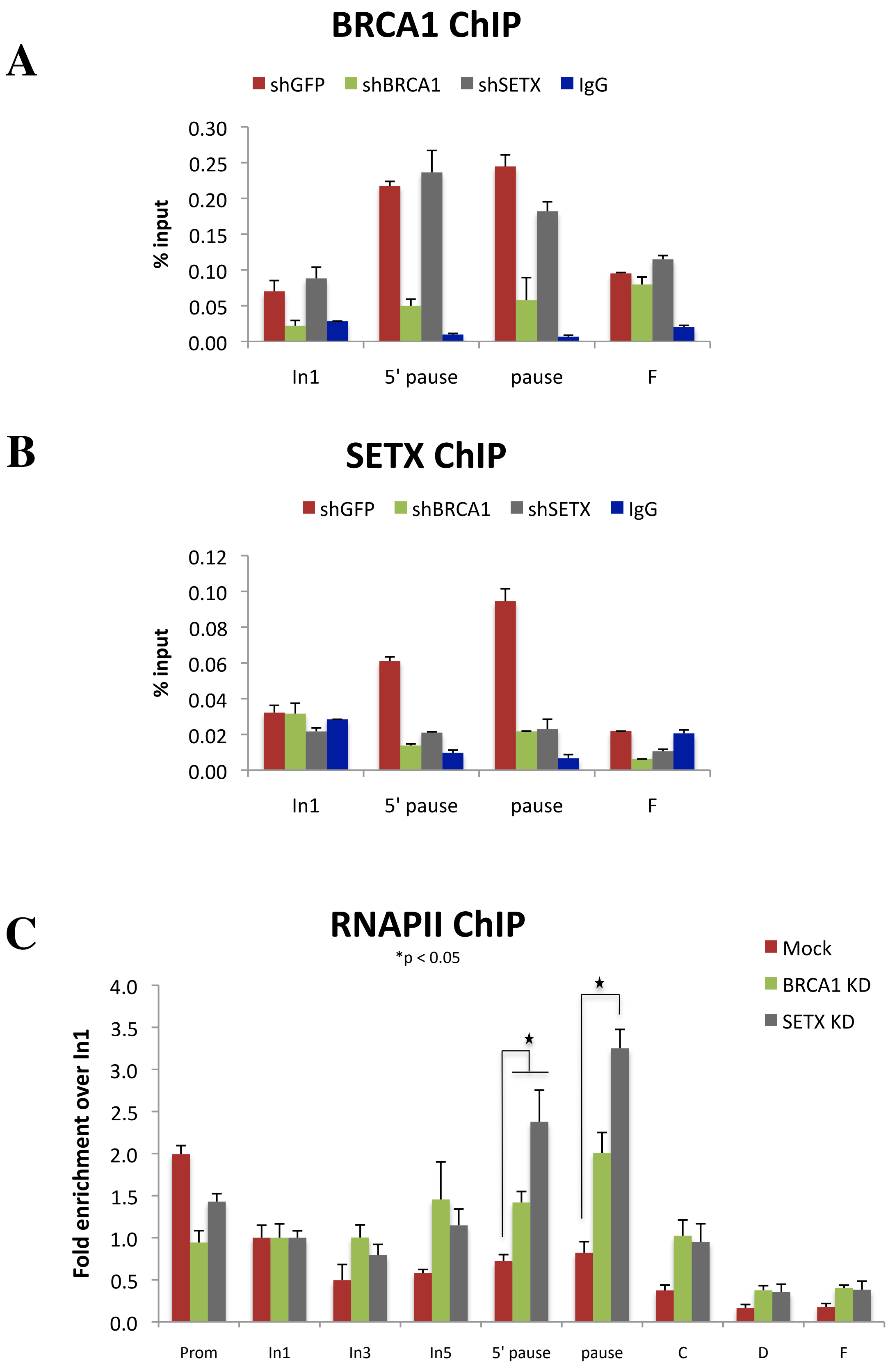

Hatchi et al. Figure S3, related to Figures 2 and 3

A

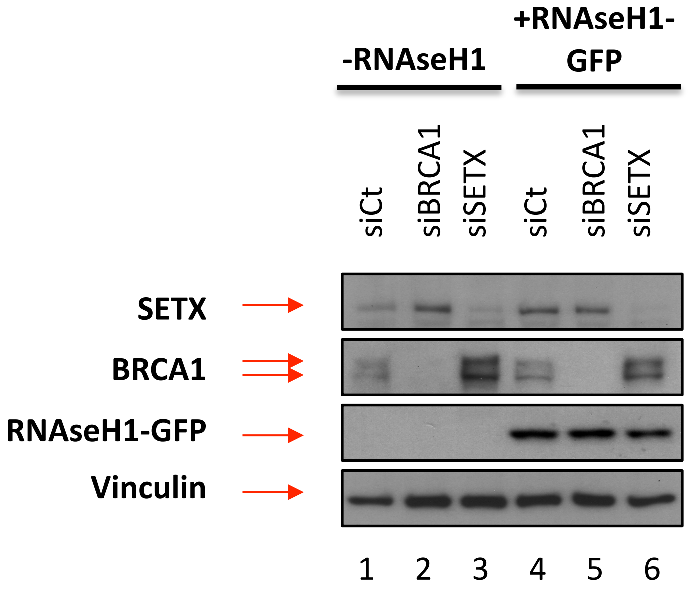


B

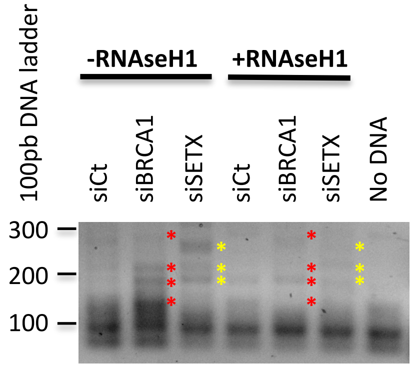


C

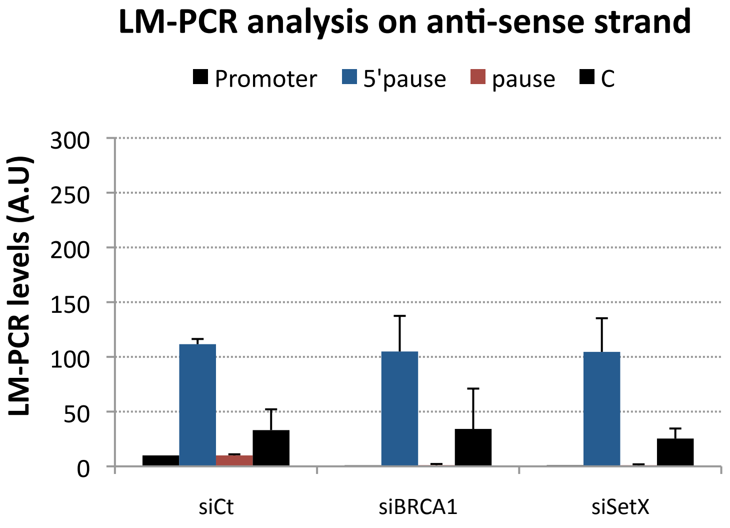

# Hatchi et al. Figure S4, related to Figure 4

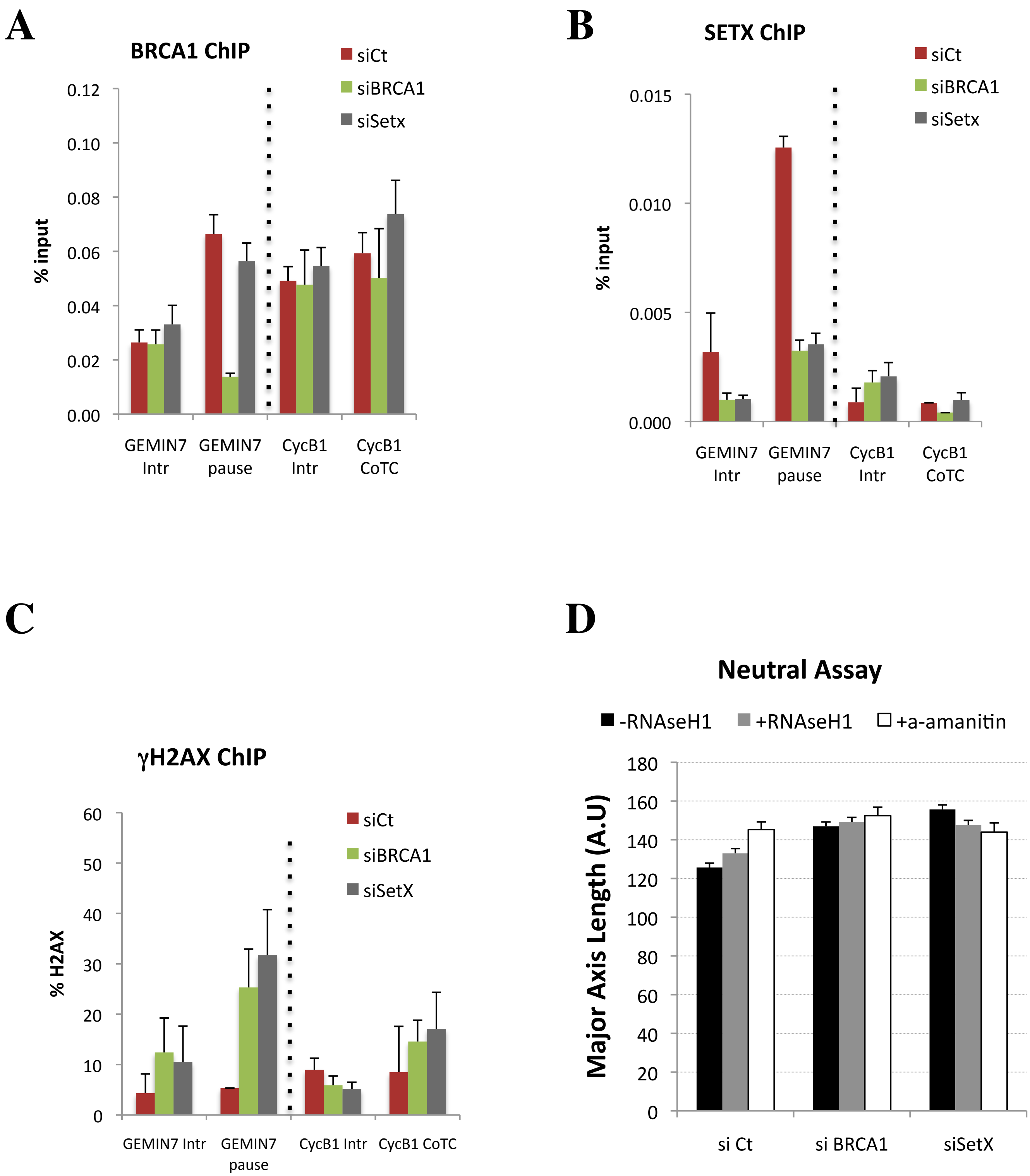

Hatchi et al. Figure S5, related to Figure 5

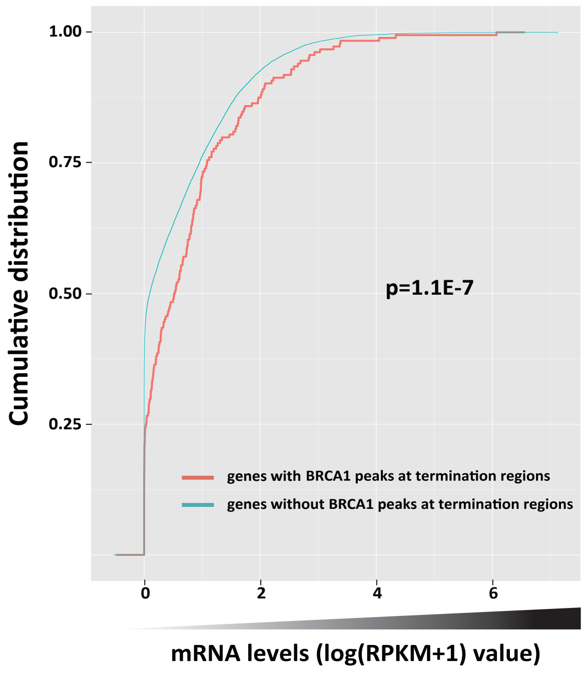

# Hatchi et al. Figure S6, related to Figure 6

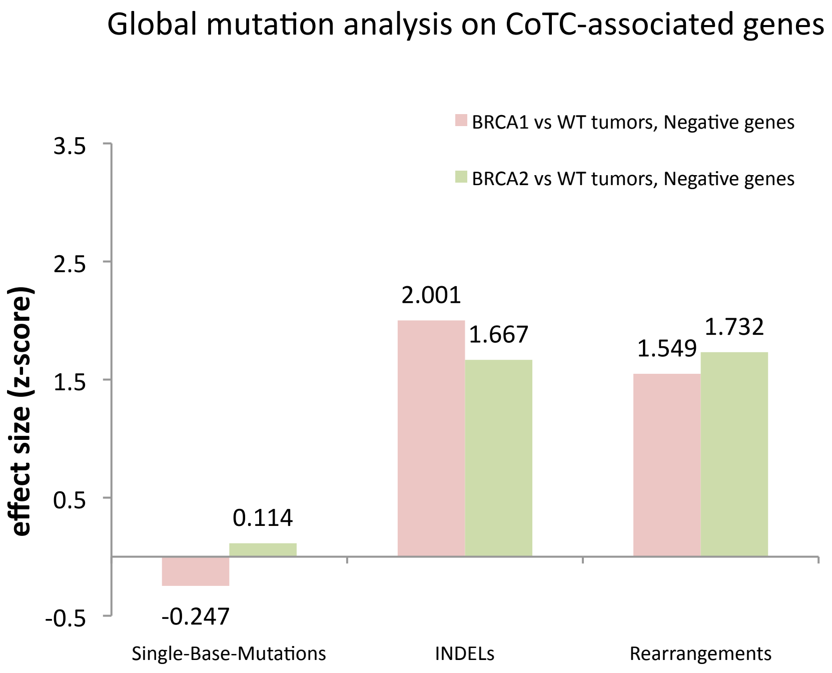

**Supplemental Figure Legends**

**Figure S1. BRCA1 and SETX antibodies, epitope validation, and GST binding results. Related to Figure 1.**

(A) Human BRCA1 p220 (referred to as BRCA1) is a multifunctional protein bearing a RING domain and paired BRCT motifs. The location of three BRCA1 monoclonal antibody epitopes is indicated. BRCA1#3 is A300-000A from Bethyl Laboratories and was used for BRCA1 ChIP assays. NLS, nuclear localization site; SCD, super-coiled domain; BRCTs, *BRCA1* C-Terminal motifs.

(B) Human SETX map. The N-terminal domain shared by human SETX and the yeast homolog Sen1 proteins is indicated in purple. The helicase domain is indicated in green. The position of the 7 helicase motifs (I–VI) is noted. The location of SETX antibody epitopes is indicated.

(C) Co-immunoprecipitation (IP) showing that the interaction between endogenous BRCA1 and endogenous SETX in HeLa cells is lost after depletion of either protein. These results confirm the specificity of the antibodies targeting BRCA1 and SETX. Input= 10% of protein used for co-IP. IgG, negative control.

(D) Schematic representation of the BRCA1 and SETX fragments produced in *E.coli* as GST fusion proteins and respectively tested for binding to SETX and BRCA1 by GST binding assays (Figure 1B).

For BRCA1, the RING domain is shown by the orange N-terminal box; exon 11 is indicated in black; and the 2 BRCT motifs, located near the C-terminus are in dark red.

The numbers represent BRCA1 amino acid residues. For SETX, the helicase domain is shown is green.

(E) BRCA1 fragment/BACH1 binding assays performed with GST-BRCA1 fragments and analyzed by immunoblotting. Blots generated with eluates of each GST-fused BRCA1 fragment were probed with anti-BACH1 antibody. Bottom panel: Relative abundance of each recombinant affinity purified fusion protein (marked with a star) visualized by SDS-PAGE after Coomassie staining. Additional bands could indicate abnormally translated or degradation products of the relevant recombinant proteins.

**Figure S2. Validation of BRCA1 and SETX recruitment to β-actin termination sites. Related to Figure 1.**

(A-C) Quantitative ChIP analyses performed with BRCA1 #3 (A), SETX #1 (B) and total RNAPII (C) antibodies or negative control IgG (blue bars) on the human β-actin gene in cells infected with lentiviruses encoding shGFP (red bars), shBRCA1 (green bars) or shSETX (grey bars). Mean values (± SD) of ChIP results derived from two independent experiments are shown. $p^* < 0.05$.

**Figure S3. Identification of R-loop-associated ssDNA breaks by LM-PCR. Related to Figures 2 and 3.**

(A) Immunoblot showing the extent of siRNA-mediated depletion of BRCA1 and SETX and the level of RNAseH1 expression in HeLa cells used for comet assays and ssDNA

break analysis. Immunoblots were performed with SETX #1 (top panel), BRCA1 SD118 (middle panel) and GFP (bottom panel) antibodies. Vinculin was used as a loading control.

(B) Detection of multiple LM-PCR products was undertaken after capture of the primer-extended and ligated fragments. This was followed by a regular PCR reaction with linker and GSP2 primers, respectively annealing to the adaptor and the far 3'-end of the β-actin gene. Analysis was performed by ethidium bromide-stained agarose gel electrophoresis. Red and yellow stars indicate the relevant PCR products that were selectively amplified in BRCA1- or SETX-depleted cells.

(C) Quantitative detection of ssDNA after LM-PCR analyses performed across several locations on the anti-sense strand of the β-actin gene in control and BRCA1 or SETX knockdown conditions. C, a 3'-end segment of the β-actin gene that lies downstream of its termination pause site.

**Figure S4. ChIP analyses at the *Gemin7* and *CyclinB1* genes and Neutral comet Assay. Related to Figure 4.**

(A-C) Quantitative ChIP analyses using antibodies directed against BRCA1 (A), SETX (B) or γH2AX/H2AX (C) performed on *Gemin7* and *CyclinB1* (CycB1) genes. Transcriptional termination of *Gemin7* and *CyclinB1* is mediated by R-loops (pause) and CoTC sequences, respectively. Intronic regions (Intr) of the two genes were used as

controls. ChIP profiles based on three independent experiments show mean values (± SD).

(D) Comet assay analysis performed in neutral buffer conditions. Histograms depict the quantitative analysis of comet tail lengths for each condition. Data are represented as the mean ± SEM from three independent experiments.

**Figure S5. Expression analysis of BRCA1 TR genes. Related to Figure 5.**

(A) Cumulative distribution plot comparing the expression of genes with (red curve) and without (blue curve) BRCA1 bound at termination regions. The level of mRNA expression increases with the value of log(RPKM+1); the gradient bar reflects the amplitude of expression (grey: low to black: high). Differences in expression levels between the red and blue groups were validated by the results of a Mann-Whitney-Wilcoxon statistical test (p=1.1E-7).

**Figure S6. Mutational analysis of CoTC-associated genes. Related to Figure 6.**

Global mutational analysis carried out in "R-loop free" CoTC genes using the complete whole-genome catalog of somatic mutations from 21 breast cancers (Nik-Zainal et al., 2012). The histogram illustrates the effect size comparison (one tailed CMH z-score) between the different tumor subgroups: BRCA1 mutant or BRCA2 mutant vs WT (non BRCA1/BRCA2 mutated) when testing the region ±4kb from a TTS.

## Supplemental Tables

**Table S1: List of BRCA1 peaks at termination regions, Related to Figure 5.** List of BRCA1 ChIP-seq peaks identified within Termination Regions (TR) showing genomic information: chromosomes, coordinates, strand, gene name associated, RNA-seq RPKM values and overlap with RNAPIISer2P ChIP-seq peaks.

**Table S2: List of the indels identified within the BRCA1 TR and CoTC genes, Related to Figure 6.** BRCA1 TR and CoTC genes were tested for mutations within 21 breast cancers containing BRCA1 or BRCA2 germline mutations. Genomic information of the indels identified are shown.

## Supplemental Experimental Procedures

**Cell lines and culture conditions.** HeLa cells and immortalized human fibroblasts (BJ-hTert) were grown at 37$^{\circ}$C in Dulbecco's Modified Eagle Medium (DMEM) supplemented with 10% fetal bovine serum (GIBCO) in a 10% $CO_2$-containing atmosphere. To secure the inhibition of RNA polymerase II, we incubated the cells overnight with 10uM $\alpha$-amanitin (Sigma) prior to harvesting the cells.

**Plasmids and knockdown experiments: siRNA transfection and lentivirus infection.** Transfection of GFP-RNAseH1 vectors (Skourti-Stathaki et al., 2011) was performed with Fugene6 transfection reagent (Promega) according to the manufacturer's instructions. The following siRNA reagents were used in this study: siBRCA1,

CAGCUACCCUUCCAUCAUA; siSETX, GCCAGAUCGUAUACAAUUA and siCt, GCGCGCUUUGUAGGAUUCG. All siRNAs were transfected into cells at 30nM (final concentration) in the presence of Lipofectamine RNAiMAX (Life Technologies). Cells were analyzed 72h post-transfection. These shRNAs were purchased from Sigma: shGFP, SHC005; shBRCA1, TRCN0000010305 and shSETX, TRCN0000051516.

**GST binding assays.** GST fragments (F1-F6 for BRCA1 and S1-S9 for SETX) have been described (Scully et al., 1997; Suraweera et al., 2009). C-terminal fragments of BRCA1 (F6N and F6C) were PCR amplified and cloned into the GST bacterial expression vector, pET42a. GST fusion proteins were generated in BL21(DE3)pLysS *E. coli* strains for 3h at 37°C after addition of 0.5 mM IPTG (isopropyl-β-D-thio-galactoside; Invitrogen). Bacteria were pelleted, resuspended in sonication buffer [50 mM Tris-HCl pH 7.6, 150 mM NaCl, 10% (v/v) glycerol, 0.5% (v/v) NP40, 1mM DTT and protease inhibitors (Roche)] and sonicated on ice. GST-tagged proteins were purified from bacterial lysates using Glutathione Sepharose 4B (GE Healthcare) and eluted from the beads with Elution Buffer (50 mM Tris pH 8, 150 mM NaCl, 10% (v/v) glycerol, 0.1% (v/v) NP40, 1mM DTT, 10mM reduced glutathione (Sigma) and protease inhibitors. Purified recombinant GST fusion proteins (2 μg of each) were bound to the Glutathione Sepharose 4B and incubated with a relevant pre-cleared WCE for 2h at 4°C with rotation. Beads were washed with immunoprecipitation buffer (detailed in Experimental Procedures supplemented with 1mM DTT and increasing concentrations of KCl (50, 100 and 150mM). Bound proteins were eluted in LDS sample buffer 1X (Invitrogen) and analyzed by immunoblotting.

**Immunoblotting.** Cells were lysed for 30 min on ice with NETN-420 [420 mM NaCl, 0.5 mM EDTA, 20 mM Tris-HCl (pH 8.0), 0.5% (v/v) Nonidet P-40 (NP-40)] and supplemented with protease inhibitors (Roche). Whole cell extracts (WCE) were centrifuged at 14,000rpm for 10min at 4ºC. Protein concentrations were measured using the BCA protein Assay Kit (Pierce). From each sample, 15ug of WCE were separated in NuPAGE® 3-8% Tris-Acetate precast gels (or 4-12% Bis-Tris for GST binding assays) and blotted onto 0.2µm nitrocellulose membranes. The following antibodies were used: mouse monoclonal anti-BRCA1 (SD118, Millipore), rabbit polyclonal anti-SETX (#1: A301-105A, Bethyl), rabbit polyclonal anti-BACH1 (B1310, Sigma) and mouse monoclonal anti-Vinculin (G-11, Santa Cruz).

**Chromatin immunoprecipitation (ChIP).** Cells were cross- linked in DMEM containing 1% formaldehyde at room temperature for 10 min with rotation. Cross-linking was stopped by the addition of glycine to a final concentration of 0.125 M. Cells were harvested and resuspended in cell lysis buffer (5mM PIPES, 85 mM KCl, 0.5% (v/v) NP40 and protease inhibitors) for 10 min on ice. Nuclei were then pelleted by centrifugation and resuspended in nuclear lysis buffer (25mM Tris-HCl pH 8, 5 mM EDTA, 1% (v/v) SDS, and protease inhibitors: Roche) for 10 min on ice. Chromatin was sheared using a cooled Bioruptor bath sonicator (Diagenode) for 20 min with 30 s pulses and 30 s pauses in order to obtain chromatin fragments with an average size of 500 bp. Quality and size of chromatin fragments was monitored by ethidium-bromide stained agarose gel electrophoresis after DNA purification. Chromatin was diluted at least 10 times into ChIP dilution buffer (1mM EDTA pH 8, 15mM Tris pH 8, 150mM NaCl

complemented with protease inhibitors). An aliquot representing 5% of the total amount of chromatin used for each IP was stored for further analysis. Chromatin (15 μg per condition) was then incubated overnight with protein A/G magnetic beads (Dynabeads, Invitrogen) coupled to the appropriate antibody: 5 μg of anti-BRCA1 (#3: A300-000A, Bethyl), 5 μg of anti-SETX (#1: A301-105A, Bethyl), 1 μg of anti–γH2AX (#05-636, Millipore), 1 μg of anti-histone H2A.X (#07-627, Millipore) and 5ug of total RNAPII (sc-9001, Santa Cruz). Immunoprecipitates were exposed to 8 serial washes for 5-10 min each on a rotating wheel at 4°C in the following buffers, complemented with protease inhibitors: 1X: 20mM Tris-HCl pH 8, 2mM EDTA, 0.1% SDS, 1% (v/v) Triton X-100 and 165mM NaCl; 1X: 20mM Tris-HCl pH 8, 2mM EDTA, 0.1% SDS, 1% (v/v) Triton X-100 and 500mM NaCl; 1X: 10mM Tris-HCl pH 8, 1mM EDTA, 1% (v/v) NP-40, 1% Na-deoxycholate and 250mM LiCl; 2X: 50mM HEPES pH 7.6, 1mM EDTA, 1% (v/v) NP-40, 0.7% Na-deoxycholate and 500mM LiCl and 3X with TE (10mM Tris.HCl pH7.5, 1mM EDTA). DNA was eluted from the magnetic beads with 0.1 M sodium carbonate and 1% (w/v) SDS. Samples were then brought to 300 mM NaCl and RNAse A (10 μg/ mL) and incubated at 65°C overnight to reverse crosslinks. DNA was purified with a PCR purification kit (Qiagen) according to the manufacturer's instructions. ChIP samples were quantified by quantitative real-time PCR using SYBR green mix (iTaq Universal, Biorad) and the primers of interest (see Table 1 for primers sequences). We analyzed the data using ABI PRISM 7900 Sequence Detection system software (Applied Biosystems). The results were calculated as %Input, representing the proportion of 100% Input (total amount of chromatin used for each IP) or as fold enrichment over a given region.

**Comet Assay.** Alkaline and neutral comet assays were performed on HeLa cells using a Single Cell Gel Electrophoresis Assay Kit (Trevigen) according to the manufacturer's instructions. 1500 cells were spotted onto each sample area. The CellProfiler software (Carpenter et al., 2006) was used for analysis and quantification of the results.

**LM-PCR and quantification of ssDNA breaks.** HeLa cells were harvested 72h after siRNA-mediated depletion of BRCA1 or SETX, and genomic DNA (gDNA) was routinely purified using a DNeasy® kit (Qiagen). The LM-1 and LM-2 oligonucleotides were annealed as described (Schlissel et al., 1993), and 300 pmoles of these adapters were phosphorylated with T4 Polynucleotide Kinase (New England Biolabs) and purified as described (Sambrook and Russell, 2006). Antisense, gene- specific primers (GSPs) for the β-actin gene were used to detect ssDNA breaks on the sense strand of the gDNA. The assay was performed using 1 μg of gDNA per sample. DNA was denatured and first annealed with biotinylated-GSP1 primers. Primer extension from the 3'-end of the β-actin gene was accomplished with Deep Vent® DNA polymerase (New England Biolabs). Then, the ds blunt end DNA was ligated to the phosphorylated asymmetric/5'-overhanged double-stranded adaptor for 2 hrs at 22°C using 2.5 U of T4 DNA ligase (New England Biolabs) and a final adaptor concentration of 1nM. Dynal M280 streptavidin-coated magnetic beads (Invitrogen) were used to capture the ligated and extended β-actin DNA fragments, according to manufacturer's instructions. The beads were resuspended in 0.1X TE, and the equivalent of 50-100 ng of purified and ligated

product were used as a template for a first round PCR reaction, using a nested gene specific primer (GSP2) and a linker primer. Samples were amplified for 12 cycles in a 3 step-protocol with Phusion High-Fidelity DNA polymerase in GC buffer (Thermo Scientific). PCR products were diluted (1/50) and processed by qPCR to amplify specific regions of interest along the β-actin gene, the 5'pause region and the pause region, itself, using the same primers that were used for ChIP experiments. We also included a negative control region, which was interrogated with primer C. It is located further downstream and outside of the primer- extended β-actin gene. As for ChIP experiments, the quantification of PCR products was analyzed with SYBR green (iTaq Universal, Biorad) using ABI PRISM 7900 Sequence Detection system software (Applied Biosystems). See Table 1 for primers sequences.

**Genomic Analyses.** Protein-coding transcripts were identified from an hg19 build based on GENCODE v10 annotations (Consortium, 2012; Harrow et al., 2012). Putative transcription termination regions (TR) were defined as a 4 kb window downstream from the transcription termination site (TTS, end of the 3' UTR). When there were multiple transcripts sharing the same gene ID, the most downstream TTS was used. Termination regions were filtered out when they overlapped promoter regions.

Using the same protein-coding transcripts as above, we defined promoter regions as being located within a sequence 1250 bp upstream and 250 bp downstream of the transcription start site (TSS=beginning of the 5' UTR). When there were multiple transcripts sharing the same gene ID, the most upstream TSS was used. To define BRCA1 peaks, we used the officially processed, HeLa-S3 BRCA1 peaks as called by

ENCODE (Consortium, 2012; Quinlan and Hall, 2010) with the SPP algorithm. The file is accessible at: http://hgdownload.cse.ucsc.edu/goldenPath/hg19/encodeDCC/wgEncodeAwgTfbsUniform/wgEncodeAwgTfbsSydhHelas3Brca1a300IggrabUniPk.narrowPeak.gz. When investigating the overlap between termination regions and BRCA1 peaks, we filtered out any BRCA1 peaks that overlapped promoters and/or transcripts.

We defined a BRCA1 peak/TR overlap as that of a termination region overlapping a BRCA1 peak, with the use of Bedtools (Consortium, 2012; Djebali et al., 2012; Quinlan and Hall, 2010). We calculated the number of instances observed between any two groups, as well as the expected instances of overlap based on the hypothetical uniform distribution of the terminations regions (TR) and BRCA1 peaks. Specifically, let $n_1$ and $n_2$ be the respective number of regions of TR and BRCA1 peaks, while $l_1$ and $l_2$ is the respective total length of regions corresponding to TR and BRCA1 peaks. Furthermore, let x be the total size of the genome. Then, the expected instances of overlaps would be approximately $E[Overlaps] = (n_1*l_2+n_2*l_1)/x$, assuming uniform distributions of TR and BRCA1 peaks over the entire genome. When using all end sites (TR) and BRCA1 peaks, our search space was the size of the genome, while the use of filtered end sites and BRCA1 peaks allowed for the reduced search space of the size of the genome without promoters.

Using the expected and observed number of overlaps, we identified statistically significant enrichments for BRCA1 peaks at putative pause sites. To test the significance of the results, we used the upper tail of the cumulative Poisson distribution, with the expected number of overlaps as the mean.

For gene expression data, RNA-Seq quantifications were downloaded from the ENCODE RNA-Seq dashboard (Consortium, 2012; Djebali et al., 2012). The RPKM scores were exported from the CSHL HeLa-S3, Long PolyA+, whole-cell extract RNA-Seq data with GENCODE v10 annotations. The quantifications were generated using the GeneGencV10IAcuff quantification view. The file is accessible at http://genome.crg.es/~jlagarde//encode/pre-DCC/wgEncodeCshlLongRnaSeq/20120220_long_quantifications_gencodev10_cufflinks_cshl_NOT_SUBMITTED/LID16633-LID16634_GeneGencV10IAcuff.gff.

With the gene expression data, we were able to compare the expression of genes whose filtered end sites did or did not overlap the filtered BRCA1 peaks. We transformed the RPKM scores to log(RPKM+1), and generated a geometric box plot (Figure 5C) and a cumulative distribution plot (Figure S4) using ggplot2 (Harrow et al., 2012; Wickham, 2009). Any gene whose putative pause site was filtered out in the previous steps was not included. The statistical significance of the variation between the two groups was tested using the non-parametric two-sided Mann-Whitney test.

To evaluate the overlap between BRCA1 peaks at end sites and RNAPIISer2P peaks, we used the RNAPIISer2P ChIP-seq data available from ENCODE at: http://hgdownload.cse.ucsc.edu/goldenPath/hg19/encodeDCC/wgEncodeSydhTfbs/wgEncodeSydhTfbsHelas3Pol2s2IggrabPk.narrowPeak.gz. To compute the p-value of BRCA1 TR and RNAPIISer2P peak overlap enrichment, we generated 10E7 random datasets having peak lengths and chromosomal distributions matching the experimental dataset. None of them had the same or a larger number of overlaps than the original file, hence $p<1.0E-7$. For the meta-analysis of co-incidence between BRCA1 peaks at ends sites and

R-loops, we used the DRIP-seq data (Ginno et al., 2012). The statistical significance of the variation between the DRIP and DRIP+RH samples was tested using the non-parametric two-sided paired Wilxocon test, as the data did not conform to normal distribution.

**Functional enrichment analyses.** Gene IDs of BRCA1 TR genes corresponding to genes with at least one BRCA1 binding site in the termination region were uploaded into Ingenuity Pathway Analysis (IPA) to search for biological and molecular functions as well as disease associations that were significantly enriched in the data set. Each p-value was determined by the Fisher's exact test and corresponds to the probability that, in a given category, we could reach the observed enrichment, or a larger one, by chance alone. Select enrichments for the BRCA1 TR gene set are shown in Figure 5F, and the full list is accessible in Table S2.

**Mutational analyses of breast cancers.** The BRCA1 TR genes as well as the CoTC "negative" genes (Nojima et al., 2013) were annotated in the hg19 build based on ENSEMBL format version 74 and scanned for each type of mutation. For each type of mutation, we computed a standardized mutation score (z-score) as follows. For each gene, we compared the observed number of mutations in BRCA1 or BRCA2 breast tumors to the number of mutations we would expect under the null hypothesis that the mutation rate in BRCA1 or BRCA2 breast tumor subgroup is equal to the mutation rate in the sporadic tumors (WT).

Then, for each subgroup of tumors, we computed the sum of the differences between observed and expected mutations across all genes; finally we standardized this sum as: z-score = (Total Observed – Total Expected) / Standard Deviation of Total Observed. This analysis produces a global standardized mutation score, where positive (or negative) values indicated a higher (or lower) number of mutations than expected under the null hypothesis. Since mutational events are rare, we computed the p-values by Monte-Carlo simulation. First, we drew 10,000 global standardized mutation scores from the exact null distribution. Under the null hypothesis, the mutation score is equal to a sum of transformed hypergeometric random variables, one for each gene. Second, we computed the p-value as the proportion of simulated mutation scores, which are greater than, or equal to, the observed mutation score.

## Sequence of DNA primers

| **Name** | **Sequence (5' →3')** |
|---|---|
| **Human β-actin gene** | |
| Prom (F) | GAGGGGAGAGGGGGTAAA |
| Prom (R) | AGCCATAAAAGGCAACTTTCG |
| In1 (F) | CGGGGTCTTTGTCTGAGC |
| In1 (R) | CAGTTAGCGCCCAAAGGAC |
| In3 (F) | TAACACTGGCTCGTGTGACAA |
| In3 (R) | AAGTGCAAAGAACACGGCTAA |
| In5 (F) | GGAGCTGTCACATCCAGGGTC |
| In5 (R) | TGCTGATCCACATCTGCTGG |
| 5'pause (F) | TTACCCAGAGTGCAGGTGTG |
| 5'pause (R) | CCCCAATAAGCAGGAACAGA |
| pause (F) | GGGACTATTTGGGGGTGTCT |
| pause (R) | TCCCATAGGTGAAGGCAAAG |
| C (F) | TGGGCCACTTAATCATTCAAC |
| C (R) | CCTCACTTCCAGACTGACAGC |
| D (F) | CAGTGGTGTGGTGTGATCTTG |
| D (R) | GGCAAAACCCTGTATCTGTGA |
| F (R) | CCATCACGTCCAGCCTATTT |
| F (F) | TGTGTGAGTCCAGGAGTTGG |
| | |
| **Human ENSA gene** | |
| Intr (F) | GCAACATGCTGGAAGAGAGAG |
| Intr (R) | AAACTACAGTGCCCCCTTAGC |
| 3' end (F) | TCTGCCTGTATTTGTGTGCTG |

| | |
|---|---|
| 3' end (R) | GCAGCCCTCGTCTGTATAATG |
| | |
| **Human Gemin7 gene** | |
| Intr (F) | GATTCTATTTGGGCCACCTATG |
| Intr (R) | GGGAGGCATCTAAACCTCATC |
| 3' end (F) | AGCTCACGCTGGTTCTTTCTT |
| 3' end (R) | GAAATTCCAAAGGCGAGAGAC |
| | |
| **Human Akirin1 gene** | |
| Intr (F) | GGCATTACAGGAGTGCTACACA |
| Intr (R) | AATGGCCTACTCAATGCCTTC |
| 3' end (F) | TGTCTGTGGCAGTTTTCACAC |
| 3' end (R) | GGCCAGCACACTTTTCTGTAA |
| | |
| **Human Cyclin B1 gene** | |
| Intr (F) | CTTGGAAGGCCCATACTGATT |
| Intr (R) | GGAGTTGATGCTTGAGCTGTT |
| 3' end (F) | GCAGGAGGGTATGCCATCTA |
| 3' end (R) | TGCTTAAGGACTGGGATATGGT |
| | |
| **LM-PCR** | |
| Long Adaptor oligonucleotides (LM1) | GCATGGATGTTTTCCCAGTCACGACGTTGTGGGGACAAGTTTGTACAAAAAAGCAGGCTTCCAAC |
| Short Adaptor oligonucleotides (LM2) | GTTGGAAGCCTGCTTTTTTGTACAAACTTGTCCCC |
| Linker primer | GCATGGATGTTTTCCCAGTCACGACGTTGT |
| GSP1 (5'-biotin) | GCAGAATCCAGACCTCAGCCCATAGCTAACCAGA |
| GSP2 | TGTCTGGATGAACAGGTAGGAAT |
| SGSP1 (5'biotin) | TAACACTGGCTCGTGTGACAA |
| SGSP2 | GAGCTGTCACATCCAGGGTC |

**Supplemental references**


Carpenter, A.E., Jones, T.R., Lamprecht, M.R., Clarke, C., Kang, I.H., Friman, O., Guertin, D.A., Chang, J.H., Lindquist, R.A., Moffat, J., et al. (2006). CellProfiler: image analysis software for identifying and quantifying cell phenotypes. Genome Biology *7*, R100.

Consortium, T.E.P. (2012). An integrated encyclopedia of DNA elements in the human genome. Nature *489*, 57–74.

Djebali, S., Davis, C.A., Merkel, A., Dobin, A., Lassmann, T., Mortazavi, A., Tanzer, A., Lagarde, J., Lin, W., Schlesinger, F., et al. (2012). Landscape of transcription in human cells. Nature *489*, 101–108.

Ginno, P.A., Lott, P.L., Christensen, H.C., Korf, I., and Chédin, F. (2012). R-Loop Formation Is a Distinctive Characteristic of Unmethylated Human CpG Island Promoters. Molecular Cell *45*, 814–825.

Harrow, J., Frankish, A., Gonzalez, J.M., Tapanari, E., Diekhans, M., Kokocinski, F., Aken, B.L., Barrell, D., Zadissa, A., Searle, S., et al. (2012). GENCODE: the reference human genome annotation for The ENCODE Project. Genome Research *22*, 1760–1774.

Nik-Zainal, S., Alexandrov, L.B., Wedge, D.C., Van Loo, P., Greenman, C.D., Raine, K., Jones, D., Hinton, J., Marshall, J., Stebbings, L.A., et al. (2012). Mutational Processes Molding the Genomes of 21 Breast Cancers. Cell *149*, 979–993.

Nojima, T., Dienstbier, M., Murphy, S., Proudfoot, N.J., and Dye, M.J. (2013). Definition of RNA Polymerase II CoTC Terminator Elements in the Human Genome. Cell Reports *3*, 1080–1092.

Quinlan, A.R., and Hall, I.M. (2010). BEDTools: a flexible suite of utilities for comparing genomic features. Bioinformatics *26*, 841–842.

Sambrook, J., and Russell, D.W. (2006) Purification of Radiolabeled Oligonucleotides by Precipitation with Ethanol. Molecular Cloning. Cshprotocols.Cshlp.org.Ezp-Prod1.Hul.Harvard.Edu.

Schlissel, M., Constantinescu, A., Morrow, T., Baxter, M., and Peng, A. (1993). Double-strand signal sequence breaks in V(D)J recombination are blunt, 5'-phosphorylated, RAG-dependent, and cell cycle regulated. Genes Dev. *7*, 2520–2532.

Scully, R., Chen, J., Plug, A., Xiao, Y., Weaver, D., Feunteun, J., Ashley, T., and Livingston, D.M. (1997). Association of BRCA1 with Rad51 in mitotic and meiotic cells. Cell *88*, 265–275.

Skourti-Stathaki, K., Proudfoot, N.J., and Gromak, N. (2011). Human Senataxin Resolves RNA/DNA Hybrids Formed at Transcriptional Pause Sites to Promote Xrn2-Dependent Termination. Molecular Cell *42*, 794–805.

Suraweera, A., Lim, Y., Woods, R., Birrell, G.W., Nasim, T., Becherel, O.J., and Lavin, M.F. (2009). Functional role for senataxin, defective in ataxia oculomotor apraxia type 2, in transcriptional regulation. Human Molecular Genetics *18*, 3384–3396.

Wickham, H. (2009). ggplot2 (Springer).
